# Selenium predominance in heavy metal mixtures is associated with hyperuricemia in Chinese adults: the role of insulin resistance

**DOI:** 10.3389/fpubh.2026.1913054

**Published:** 2026-08-27

**Authors:** Jing-Fu Lai, Ya-Qin Zhang, Yun-Ting Zhang, Yu He, Hai-Xin Tu, Qi Yang, Wen-Wen Bao, Li-Xia Liang, Jing-Wen Huang, Zhao-Huan Gui, Li-Wen Hu, Li-Zi Lin, Yang Zhou, Bin Xu, Guang-Hui Dong, Hui-Dong Song, Feng-Hong Wu, Ru-Qing Liu

**Affiliations:** 1 Joint International Research Laboratory of Environment and Health, Ministry of Education, Guangdong Provincial Engineering Technology Research Center of Environmental Pollution and Health Risk Assessment, Department of Occupational and Environmental Health, School of Public Health, Sun Yat-sen University, Guangzhou, China; 2 Laboratory of Occupational Environment and Health Effects, Guangzhou Prevention and Treatment Center for Occupational, Diseases, Department of Gastroenterology, Guangzhou Twelfth People's Hospital, Guangzhou, China; 3 Department of Public Health, Nanning Center for Disease Control and Prevention, Nanning, China

**Keywords:** benchmarks dose, heavy metals, insulin resistance, multi-pollutant model, uric acid

## Abstract

**Introduction:**

Emerging studies indicate exposure to heavy metals is linked with uric acid levels and hyperuricemia risk. However, the combined effects of heavy metals and the underlying mechanisms remain poorly characterized.

**Methods:**

In this community-based cross-sectional study, plasma concentrations of 20 heavy metals and serum uric acid were measured in 1,312 adults in China. Triglyceride-glucose (TyG) index, a biomarker of insulin resistance, was also calculated. Generalized linear regression model, bayesian kernel machine regression, weighted quantile sum, and quantile G-computation were used to explore the associations between single or co-exposure to heavy metals and serum uric acid levels (SUA), as well as hyperuricemia (HUA). We also explored the mediating role of TyG in these associations. And then, benchmark dose lower bound (BMDL) of selenium (Se) was calculated using benchmark dose model.

**Results:**

In the single pollutant model, Se was positively associated with SUA (β = 0.18, 95% CI: 0.04, 0.32) and HUA (OR = 1.51, 95% CI: 1.08, 2.11). Three multi-pollutant models all revealed positive associations between metal mixtures and SUA as well as HUA, with Se being the primary contributor. Furthermore, TyG significantly mediated the associations of Se with SUA and HUA, with the mediated proportions of 47.85% and 34.72%, respectively. BMDL of Se was 118.70–233.12 μg/d based on HUA.

**Discussion:**

In conclusion, metal mixtures had positive associations with SUA and HUA. Insulin resistance might be a crucial mediated pathway between Se and outcomes. Our estimated point of departure (POD) for Se was lower than those recommended by other studies.

## 1 Introduction

Hyperuricemia, defined as excessive serum uric acid concentrations, represents a metabolic disturbance implicated in the pathogenesis and progression of multiple non-communicable diseases, including chronic kidney disease, gout and cardiovascular disease (1, 2). Hyperuricemia represents a substantial global disease burden, exhibiting notably high prevalence rates in the United States and China (1, 3, 4). For example, a national study reported that 20.1% of adults in the United States had hyperuricemia in 2016 (5). Data from two national surveys show that the incidence of hyperuricemia among China has increased, climbing from 11.1% in 2016 to 17.4% in 2019 (6). Therefore, it's necessary to elucidate the risk factors and underlying mechanisms. Emerging research suggests a possible link between environmental pollution and elevated uric acid levels, with heavy metals potentially being significant contributing factors (7–12).

Heavy metals are ubiquitous environmental pollutants that enter the human body through a number of pathways, such as the respiratory and digestive systems (13–15). While accumulating evidence suggests their potential disruption of purine catabolism leading to elevated uric acid levels (16, 17), current epidemiological findings exhibit substantial heterogeneity. For example, the Wuhan-Zhuhai cohort from China (*n* = 3,909) demonstrated a significantly positive correlation between co-exposure to 8 urinary metals and serum uric acid levels through Bayesian Kernel Machine Regression (BKMR) and Weighted Quantile Sum (WQS) models (18). Similarly, Wu et al. identified a positive association between blood co-exposure to five metals and elevated uric acid concentrations in 14,871 U.S. adults using WQS regression (19). However, a United States study of 3,926 participants found no significant association between urinary co-exposure to eight metals and serum uric acid concentrations using BKMR (20). These discrepancies may stem from differential biomarker matrices (urinary vs. blood metals), varying metal combinations in mixture models, and population-specific characteristics. Given the inconsistencies across studies and the limited subsets of metals in multi-pollutant models, accurately capturing the complexity of real-world human exposure to multiple metals remains challenging. Therefore, further investigation is warranted to investigate the relationship between various heavy metals and serum uric acid.

It is essential to investigate the potential biological mechanisms through which metals affect uric acid levels and hyperuricemia for elucidating the toxicity profiles of metals. Previous studies have reported that imbalanced heavy metals can affect insulin sensitivity and lead to insulin resistance (21–23). Further research has shown that insulin resistance is a pathophysiological mechanism of hyperuricemia (24, 25). For example, Facchini et al. have found that greater insulin resistance correlated positively with increased serum uric acid levels (26). Thus, insulin resistance may serve as a critical link in the mechanistic pathways connecting metals to hyperuricemia. However, to date, current evidence lacks quantification of insulin resistance as a mediating pathway linking heavy metal exposure to uric acid level.

Furthermore, certain metals, such as selenium, may pose significant health risks when present in the human body at either elevated or deficient concentrations. For example, excessive selenium exposure has been causally linked to the induction of acute selenosis in human population (27). However, the point of departure (POD), which can help create a “safe dose” (such as reference dose), for such metals based on observed human adverse outcomes is still lacking in China (Supplementary Table S1).

We aimed to investigate the associations of individual heavy metal and metals mixtures with SUA levels and HUA among Chinese adults, to evaluate whether insulin resistance assessed by the TyG index mediated these associations, and to identify the dominant metal in the mixture and estimate its point of departure using benchmark dose modeling.

## 2 Materials and methods

### 2.1 Study population

This study was a community-based cross-sectional study conducted in Guangzhou, a coastal megacity in southeastern China, examining associations between adult environmental exposures and health outcomes, with methodological details published previously (28, 29). In brief, between November 2018 and August 2019 three districts (Conghua, Yuexiu, and Panyu) from the eleven administrative districts of Guangzhou were randomly selected and 1,524 residents were selected to participant in this study based on the principle of cluster random sampling. Participants were excluded according to the following criteria: (1) age < 18 (*n* = 78); (2) missing basic questionnaire information (*n* = 12); (3) absence of blood sample measurement (*n* = 122). Finally, a total of 1,312 participants aged 18~88 were enrolled in this study, with an effective response rate of 86.1% (Supplementary Figure S1).

Information on demographic characteristics, socioeconomic status, smoking, drinking, and physical activity was collected by trained investigators using standardized questionnaires. Height and weight were measured using standardized protocols. Fasting venous blood samples were collected in the morning after an overnight fast, and were centrifuged and stored at −80 °C until analysis. Plasma samples were used for measurement of heavy metals, and serum samples for measurement of uric acid, creatinine, fasting glucose, and triglycerides. All enrolled individuals provided written informed consent prior to study commencement, and the protocol for this study has been approved by the Human Research Committee of Sun Yat-sen University.

### 2.2 Measurement of plasma heavy metals concentration

The contents of 20 metals in plasma samples were quantified by inductively coupled plasma mass spectrometry (ICP-MS, Thermfer Fischer Technologies, iCAPRQ), including selenium (Se), aluminum (Al), antimony (Sb), arsenic (As), iron (Fe), copper (Cu), zinc (Zn), tin (Sn), beryllium (Be), vanadium (V), manganese (Mn), strontium (Sr), cobalt (Co), nickel (Ni), yttrium (Y), cadmium (Cd), cerium (Ce), thallium (Tl), lead (Pb), and chromium (Cr). The percentile concentrations, limit of detection (LOD), and detectable ratios for the quantified heavy metals were presented in Supplementary Table S2. Detailed methods for sample extraction, analysis, and quality control were provided in Method S1 and Method S2. For all metals analyzed, LOD ranged from 0.001 to 0.561 μg/L, and LOD/√2 was used in place of the concentration for values below the LOD (30). Although some elements (Se and As) are chemically classified as metalloids, the term “heavy metals” is used here collectively for metals and metalloids, following common practice in environmental epidemiology (18, 31, 32).

### 2.3 Estimated daily intake of Se

Estimated daily intake (EDI) of selenium was estimated from plasma selenium concentration according to previously published biomarker-based and model-based approaches (33–35). Because these approaches were developed in adult populations and plasma selenium is an appropriate biomarker of internal selenium status, we considered this conversion acceptable for approximating selenium intake in our adult study population.

EDI of selenium was calculated by the formulas as follow:

$$\begin{array}{l} \log Y=a\log X+b \end{array}$$ (1)

$$\begin{array}{l} \tau =\frac{CV}{IF} \end{array}$$ (2)

Firstly, we calculated the blood Se concentration based on Equation 1, where Y represents the plasma Se concentration (μg/L), X represents the blood Se concentration (μg/L), a represents the slope factor value (0.749) and b represents intercept value (0.387), as reported in a previous study (33). Secondly, we calculated the EDI based on Equation 2, where τ is the reciprocal of the biological half-life constant of selenium (17 days) (34), C is the concentration of Se in blood (μg/L), V is the apparent blood volume of the human body (estimated at 5.5 L), I is the EDI (μg/d) and F is the fractional absorption of ingested selenium into systemic circulation (estimated at 50%), respectively (35). EDI was calculated to characterize the selenium exposure distribution in our study population. This distribution was subsequently compared to the derived benchmark dose lower bound (BMDL) to assess the potential public health implication of the exposure levels observed.

### 2.4 Examination of serum uric acid and definition of hyperuricemia

Serum uric acid (SUA) and serum creatinine (Scr) were quantitatively detected using a Roche automatic analyzer manufactured in Mannheim, Germany. Hyperuricemia (HUA) is defined as a SUA concentration greater than 360 μmol/L in women and 420 μmol/L in men (36).

### 2.5 Mediation factor

The triglyceride-glucose (TyG) index is a simple and reliable indicator that is easily obtained in clinical practice and can be used as a common indicator of insulin resistance (37, 38). Evidence from modern research shows that the TyG index can effectively detect insulin resistance in various populations (39–41). TyG index equals to Ln (fasting triglycerides × fasting glucose/2) (37). In the equation, fasting triglycerides and fasting glucose were expressed as mg/dL. Fasting triglycerides are measured using a Roche automatic analyzer, while fasting glucose is measured using an automatic biochemical analyzer.

### 2.6 Covariates

According to the previous literature (8, 42) and the directed acyclic graph (DAG) analysis (Supplementary Figure S2), we identified covariates for this study, including sex (men, women), body mass index (BMI) (kg/m^2^), age (years), serum creatinine (mg/dL), physical activity (< 1 time/week, 1–2 times/week, ≥3 times/week), annual income (< 30,000 Chinese Yuan, CNY; 30,000–100,000 CNY; >100,000 CNY), education level (< high school, ≥high school), drinking status (yes, no) and smoking status (yes, no).

According to the World Health Organization (WHO) standards, BMI values 25 kg/m^2^ or higher were considered as overweight, and those 30 kg/m^2^ or more were considered as obese.

### 2.7 Statistical analysis

#### 2.7.1 Basic information analysis

Bartlett's and Shapiro-Wilk tests were used to evaluate variance homogeneity and distribution normality. Variables with a normal distribution are reported as mean ± standard deviation (mean ± SD), whereas other variables were displayed as median with the first and third quartiles [median (Q1, Q3)]. Categorical variables were presented in the form of numbers (percentages). Plasma heavy metal concentrations underwent natural logarithmic transformation (ln-transformed). Their interrelationships were quantified using Spearman's rank correlation coefficients.

#### 2.7.2 Single metal exposure analysis

GLM models were used to explore the relationships between individual plasma metals and SUA, as well as the ORs of HUA. The models used were as follows: (1) crude model; (2) adjusted for physical activity, education, BMI, serum creatinine, annual income, sex, smoking, drinking and age.

To consider the impact of various factors on the association between metals and SUA, we performed a stratified analysis using the GLM. These analyses were stratified by age ( ≤ 65 years old, > 65 years old), sex (Men, Women), BMI (Normal, Overweight/Obesity), drinking (No, Yes) or smoking (No, Yes). Se, Sn and Cr were selected for stratified analysis which showed statistical significance in the adjusted model as mentioned above. A two-sample *t*-test was calculated to represent *P*-value for group differences (43).

#### 2.7.3 Multi-metals model

We used Bayesian Kernel Machine Regression (BKMR), Weighted Quantile Sum (WQS), and quantile G-computation (qg-comp) models to test the combined associations of metal mixtures. The main pollutants in three multi-metals models were screened using a Venn diagram. Briefly, the BKMR method used a hierarchical approach for variable selection, and a combined exposure-outcome relationship is formed after 10,000 interactions of fitting using a Markov Chain Monte Carlo (MCMC) sampler. Heavy metals are divided into two groups, distinguishing between metalloids and metals (31, 44), with As, Se as group 1, Al, Fe, Cu, Zn, Sr, Sn, Sb, Be, V, Mn, Co, Ni, Y, Cd, Ce, Tl, Pb and Cr as group 2. With the other metal holding its median level constant, the relationship between each metal and the results was displayed. The relative importance of metals is determined by calculating group posterior inclusion probabilities (group PIP) and conditional posterior inclusion probabilities (cond PIP) (45). A PIP threshold of 0.5 is conventionally applied to evaluate variable significance. In the WQS model, we constructed WQS indexes by bootstrapping with 10,000 iterations which represented the total associations of heavy metals mixture. The data were randomly divided into 40% as the training dataset and 60% as the validation datasets. Metals weighing more than 0.05 (1/20) are believed to have a significant impact (46). In addition, we constructed two separate WQS regression models with positive and negative constraints, respectively, to independently identify heavy metal mixtures associated with positive and negative associations with the outcome. In qg-comp model, it calculated the cumulative metals exposure associations whether linear or not, which was accord with the actual exposure in our daily lives (47). Moreover, the impact of each metal is quantified by its weight. Predicted weights >0.05 (1/20) demonstrated significant associations with the qg-comp score. The mutual validation of these three multi-pollutant models ensured the robustness and reliability of the results.

#### 2.7.4 Mediation analysis

According to the mediator's criterion (48), in addition to the positive correlation between X (metals) and Y (SUA and HUA), statistically significant correlations between X and M (TyG) and M and Y should be noted. Thus, we used GLM to investigate the relationships between metals and TyG, as well as between TyG and SUA and the risk of HUA. If these requirements were met, the mediation effect model would be used to determine the mediated fraction (%) of TyG in the connections between metals, SUA, and HUA in single models. The indirect and total associations were estimated using structural equation modeling, from which the mediation ratio was derived.

#### 2.7.5 Sensitivity analysis

In order to verify the reliability of our research results, we conducted a series of tests to assess its stability. Firstly, multiple linear regression analysis was used to study the association between uric acid levels and metals concentration and metals concentration were separated into quartile categories [Q1 (reference), Q2, Q3, and Q4] to determine the *P*-value for trend. Secondly, to account for potential clustering by recruitment region, we additionally fitted mixed-effects models with district specified as a random intercept. Third, taking into account the impact of comorbidities, we included hypertension, dyslipidemia and diabetes in the regression model for analysis. Finally, since uric acid levels were influenced by the chronic kidney disease (CKD), we excluded participants with CKD (49). CKD participants were identified using the diagnostic criteria of the estimated glomerular filtration rate (eGFR) of < 60 mL/min per 1.73 m^2^ (CKD) (50). The method of eGFR calculation was shown in the Method S3.

#### 2.7.6 Benchmark dose estimation

**Figure 1:**
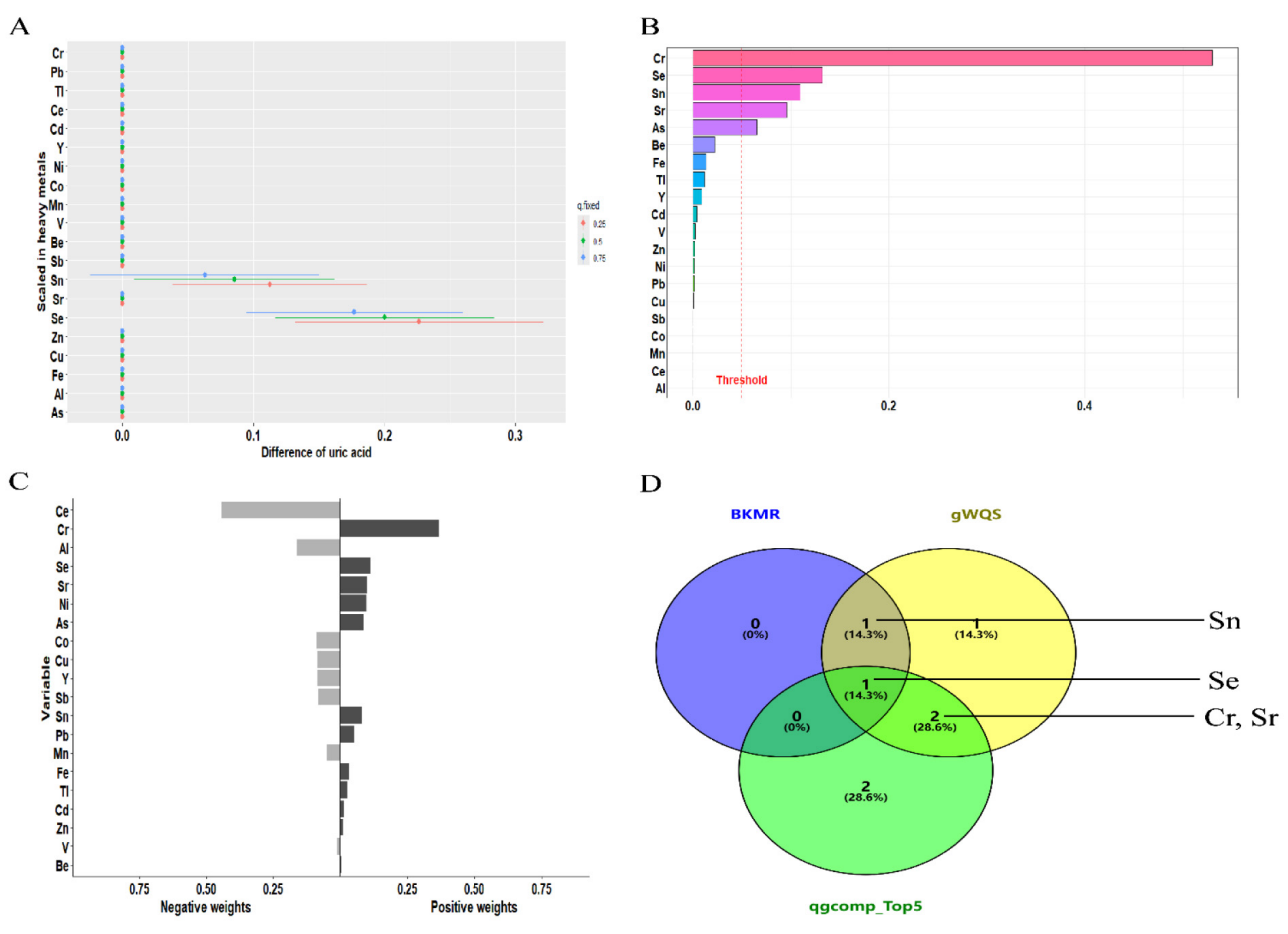
Multi-pollutant models showed that the effect of heavy metals on the change of uric acid in total population (*n* = 1,312). **(A)** Single metal-exposure effect (95% CI) on uric acid when other pollutants were fixed at 25th, 50th, and 75th percentile in BKMR models. **(B)** The individual contributor of metals mixture to the change of uric acid in positive WQS regression. **(C)** Estimated weight for the association between exposure to metals mixtures and uric acid in qg-comp model. **(D)** Venn diagram for three muti-pollutant models. All models were adjusted for age, sex, BMI, serum creatinine, annual income, education, drinking, smoking, and physical activity. BKMR, Bayesian kernel machine regression; WQS, weighted quantile sum; qg-comp, quantile G-computation.

Se was identified as a key pollutant in all three models (Figure 1), we accordingly established its BMDL. Based on the EDI of Se, participants were divided into 10 groups (0–177.99, >177.99–257.91, >257.91–298.21, >298.21–332.08, >332.08–365.43, >365.43–401.62, >401.62–443.43, >443.43–501.01, >501.01–626.78, >626.78μg/d). The dose-response associations between EDI and SUA as well as HUA were assessed using the chi-square linear trend test. In this study, we used a web-based system (https://benchmarkdose.com/) to perform Bayesian Baseline Dose Estimation (BBMD) (51). Eight continuous functions and eight dichotomous functions were selected to model the input dose-response data for SUA levels and HUA, respectively. Posterior prediction *P*-value (PPP) was implemented to evaluate the goodness-of-fit of the models, with values between 0.05 and 0.95 reflecting a satisfactory fit (51). To achieve cross-model comparisons, the algorithm assigns a weighting factor to each model and applies the weighted combined results of the eight candidate models to arrive at the baseline dose (BMD) and BMDL at baseline response (BMR) levels of 5 and 10%, respectively.

## 3 Result

### 3.1 Characteristics of participants and exposure distribution

Tables 1, 2 detail the demographic information of the participants and the distribution of exposure by HUA status. The mean age of participants was 55 ± 15.32 years, of which 60.7% were women. The prevalence of HUA in this study was 39.56%. Individuals with HUA are significantly older (57.3 vs. 53.6 years, *P* < 0.001), higher BMI (24.81 kg/m^2^ vs. 23.13 kg/m^2^, *P* < 0.001), the TyG index was higher (8.91 vs. 8.57, *P* < 0.001), and serum creatinine levels were also increased (1.00 mg/dl vs. 0.87 mg/dl, *P* < 0.001). Compared with participants of non-hyperuricemia, the median concentrations of Se, Sr, Sn, As, Fe and Cr were higher in the individuals with HUA (*P* < 0.05).

**Table 1:** Characteristics of participants distribution (*n* = 1312).

Variables^a^	Total (*n* = 1,312)	Hyperuricemia (*n* = 519)	Non hyperuricemia (*n* = 793)	*P* value
Age (years)	55.09 ± 15.32	57.34 ± 15.17	53.62 ± 15.26	**< 0.001**
Sex, Women	797 (60.7%)	294 (56.6%)	503 (63.4%)	**0.015**
BMI (kg/m^2^)	23.80 ± 3.62	24.81 ± 3.59	23.13 ± 3.49	**< 0.001**
Education ≥ High school	760 (57.9%)	283 (54.5%)	477 (60.2%)	0.050
Drinking	184 (14.0%)	85 (16.4%)	99 (12.5%)	0.064
Smoking	220 (16.8%)	89 (17.1%)	131 (16.5%)	0.733
Annual income (CNY)				0.947
< 30,000	60 (4.6%)	24 (4.6%)	36 (4.5%)	
30,000–100,000	540 (41.2%)	212 (40.8%)	328 (41.4%)	
>100,000	712 (54.3%)	283 (54.5%)	429 (54.1%)	
Physical activity (per week)				0.773
< 1 time	161 (12.3%)	66 (12.7%)	95 (12.0%)	
1–2 times	249 (19.0%)	103 (19.8%)	146 (18.4%)	
≥3 times	902 (68.8%)	350 (67.4%)	552 (69.6%)	
Serum creatinine (mg/dL)	0.92 ± 0.37	1.00 ± 0.30	0.87 ± 0.41	**< 0.001**
Uric acid (mg/dL)	4.14 ± 1.12	5.18 ± 0.83	3.46 ± 0.66	**< 0.001**
Blood sugar (mg/dL)	101.04 ±30.47	104.14 ±30.25	99.00 ±30.47	**0.003**
Triglycerides (mg/dL)	148.05 ±129.60	177.48 ±157.75	128.79 ±102.89	**< 0.001**
TyG	8.71 ±0.65	8.91 ±0.65	8.57 ±0.62	**< 0.001**
CKD	158 (12.0%)	73 (46.2%)	85 (53.8%)	**0.045**
Dyslipidemia	561 (42.8%)	279 (53.8%)	282 (35.6%)	**< 0.001**
Hypertension	357 (27.2%)	198 (38.2%)	159 (20.1%)	**< 0.001**
Diabetes	125 (9.6%)	64 (12.4%)	61 (7.7%)	**0.007**

BMI, body mass index; TyG, Triglycerides and glucose index; CNY, Chinese Yuan; CKD, chronic kidney disease. ^a^Continuous variables are presented as mean ± standard deviation and categorical variables are presented as numbers (percentage). Heavy metals were presented as median (Q1, Q3). Q1, quantile 1 ( ≤ 25th); Q4, quantile 4 (≥75th). Bold text indicates that group comparison between hyperuricemia with non-hyperuricemia were statistically significant at *P* < 0.05 based on Bartlett's test and chi-squared test.

**Table 2:** Distribution of heavy metal concentrations (*n* = 1312).

Variables	Total (*n* = 1,312)	Hyperuricemia (*n* = 519)	Non hyperuricemia (*n* = 793)	*P* value
**Heavy metals (ng/mL), median (Q1, Q3)**				
Se	49.81 (40.93, 60.05)	53.00 (41.59, 63.97)	48.10 (40.59, 58.09)	**< 0.001**
Sn	0.20 (0.08, 0.42)	0.22 (0.10, 0.62)	0.19 (0.07, 0.37)	**0.009**
Cr	0.37 (0.37, 18.35)	0.37 (0.37, 21.49)	0.37 (0.37, 14.61)	**0.019**
As	0.77 (0.38, 1.69)	0.89 (0.41, 1.92)	0.71 (0.35, 1.51)	**0.007**
Fe	790.75 (568.40, 1038.97)	798.10 (573.19, 1110.24)	781.00 (561.60, 1015.00)	**0.034**
Cu	945.23 (726.32, 1145.89)	935.46 (682.80, 1163.60)	954.01 (740.26, 1142.53)	0.900
Zn	882.89 (700.34, 1046.99)	897.40 (668.10, 1076.96)	872.39 (712.84, 1017.97)	0.084
Al	25.98 (8.36, 49.94)	23.68 (7.74, 50.13)	27.76 (8.89, 49.59)	0.421
Sr	29.47 (17.14, 41.26)	30.82 (17.46, 44.08)	29.03 (16.92, 39.03)	**0.021**
Sb	1.62 (1.39, 1.94)	1.64 (1.40, 1.94)	1.61 (1.39, 1.94)	0.431
Be	0.06 (0.06, 0.28)	0.06 (0.06, 0.30)	0.06 (0.06, 0.28)	0.970
V	0.05 (0.01, 0.16)	0.06 (0.01, 0.17)	0.05 (0.01, 0.16)	0.475
Mn	0.56 (0.26, 1.14)	0.57 (0.29, 1.12)	0.55 (0.24, 1.18)	0.236
Co	0.04 (0.02, 0.08)	0.04 (0.02, 0.08)	0.04 (0.02, 0.08)	0.182
Ni	0.17 (0.17, 1.30)	0.17 (0.17, 1.54)	0.17 (0.17, 1.20)	0.308
Y	0.01 (0.01, 0.02)	0.01 (0.01, 0.02)	0.01 (0.01, 0.01)	0.991
Cd	0.04 (0.01, 0.07)	0.04 (0.02, 0.08)	0.04 (0.01, 0.07)	0.058
Ce	0.02 (0.02, 0.12)	0.02 (0.02, 0.12)	0.02 (0.02, 0.11)	0.829
Tl	0.05 (0.03, 0.06)	0.05 (0.03, 0.06)	0.05 (0.03, 0.06)	0.220
Pb	0.78 (0.02, 2.00)	0.76 (0.02, 2.11)	0.78 (0.02, 1.93)	0.606

Q1: quantile 1 ( ≤ 25th); Q4: quantile 4 (≥75th). Al, aluminum; Sb, antimony; As, arsenic; Fe, iron; Cu, copper; Zn, zinc; Sn, tin; Be, beryllium; V, vanadium; Mn, manganese; Sr, strontium; Co, cobalt; Ni, nickel; Y, yttrium; Cd, cadmium; Ce, cerium; Tl, thallium; Pb, lead; Cr, chromium; Se, selenium. Bold text indicates that group comparison between hyperuricemia with non-hyperuricemia were statistically significant at *P* < 0.05 based on Bartlett's test and chi-squared test.

Spearman's rank correlation analysis was used to evaluate pairwise correlations among the measured metal concentrations, as the metal concentrations were not normally distributed. As shown in Supplementary Figure S3, the correlation coefficients ranged from −0.75 to 0.82. According to the predefined thresholds, several metals showed strong correlations. Strong positive correlations were observed between Cu and Zn (*r* = 0.82), Sn and Cr (*r* = 0.79), Cu and Tl (*r* = 0.74), Zn and Tl (*r* = 0.73), and Be and Sn (*r* = 0.71). In contrast, strong negative correlations were observed between Sn and Tl (*r* = −0.75), Cu and Sn (*r* = −0.72), Tl and Cr (*r* = −0.71), Cu and Cr (*r* = −0.68), and Zn and Sn (*r* = −0.66).

### 3.2 Associations between individual metal and SUA or HUA

In both the crude and adjusted models, Se and Sn were positively correlated with SUA levels and the risk of HUA (Table 3, Table 4), while Cr was positively correlated with uric acid levels only in the crude and adjusted models (Table 3). In the adjusted model, each log unit increase in Se, Sn and Cr increased SUA by 0.18 mg/dL (95% CI: 0.04, 0.32), 0.06 mg/dL (95% CI: 0.03, 0.08) and 0.04 mg/dL (95% CI: 0.02, 0.06), respectively (Table 3). Each log unit increase in Se and Sn increased the risk of hyperuricemia by 51% (OR = 1.51, 95% CI: 1.08, 2.11) and 7% (OR = 1.07, 95% 95% CI: 1.01, 2.13), respectively (Table 4).

**Table 3:** Mean difference (β) and 95% confidence intervals (95% CI) in serum uric acid (mg/dL) per log heavy metals (ng/mL) in generalized linear regression models (*n* = 1312).

Heavy metals	Crude model		Adjusted model^**a**^	
	β **(95% CI)**	***P***	β **(95% CI)**	***P***
Se	**0.34 (0.18, 0.50)**	**< 0.001**	**0.18 (0.04, 0.32)**	**0.02**
Sn	**0.07 (0.04, 0.10)**	**< 0.001**	**0.06 (0.03, 0.08)**	**< 0.001**
Cr	**0.05 (0.03, 0.07)**	**< 0.001**	**0.04 (0.02, 0.06)**	**< 0.001**
As	0.03 (−0.02, 0.07)	0.23	0.02 (−0.02, 0.06)	0.30
Fe	**0.23 (0.12, 0.34)**	**< 0.001**	0.09 (−0.02, 0.19)	0.14
Cu	**−0.14 (−0.25**, **−0.03)**	**0.01**	−0.08 (−0.18, 0.02)	0.11
Zn	−0.02 (−0.14, 0.11)	0.8	0.03 (−0.08, 0.15)	0.64
Al	−0.01 (−0.04, 0.02)	0.54	−0.01 (−0.04, 0.01)	0.19
Sr	0.02 (−0.01, 0.05)	0.17	0.01 (−0.01, 0.04)	0.31
Sb	0.11 (−0.04, 0.25)	0.15	−0.03 (−0.16, 0.10)	0.61
Be	0.02 (0.00, 0.04)	0.05	0.01 (0.00, 0.03)	0.10
V	−0.02 (−0.06, 0.03)	0.47	0.00 (−0.05, 0.04)	0.63
Mn	**0.06 (0.01, 0.10)**	**0.03**	0.02 (−0.02, 0.07)	0.34
Co	−0.06 (−0.13, 0.01)	0.07	−0.02 (−0.08, 0.04)	0.43
Ni	−0.00 (−0.05, 0.04)	0.89	0.01 (−0.03, 0.05)	0.97
Y	−0.01 (−0.11, 0.08)	0.81	−0.07 (−0.15, 0.02)	0.09
Cd	0.03 (−0.03, 0.10)	0.29	0.01 (−0.05, 0.07)	0.82
Ce	−0.00 (−0.05, 0.04)	0.86	−0.02 (−0.06, 0.02)	0.15
Tl	−0.02 (−0.06, 0.02)	0.27	−0.03 (−0.06, 0.01)	0.15
Pb	0.01 (−0.02, 0.04)	0.45	0.01 (−0.02, 0.03)	0.91

Bold text indicates that the associations were statistically significant at *P* < 0.05. This table presents the analysis results based on the single pollutant model. That is, the correlation of each heavy metal was obtained by treating it (in the form of per log10 increase) as the main exposure variable and placing it alone in a regression model. Al, aluminum; Sb, antimony; As, arsenic; Fe, iron; Cu, copper; Zn, zinc; Sn, tin; Be, beryllium; V, vanadium; Mn, manganese; Sr, strontium; Co, cobalt; Ni, nickel; Y, yttrium; Cd, cadmium; Ce, cerium; Tl, thallium; Pb, lead; Cr, chromium; Se, selenium. ^a^Models were adjusted for age, sex, BMI, annual income, education, drinking, smoking, physical activity, and serum creatinine.

**Table 4:** Odds ratio (OR) and 95% confidence intervals (95% CI) for hyperuricemia per log heavy metals (ng/mL) in generalized linear regression models (*n* = 1312).

Heavy metals	Crude model		Adjusted model^a^	
	**OR (95% CI)**	***P***	**OR (95% CI)**	***P***
Se	**1.75 (1.29, 2.40)**	**< 0.001**	**1.51 (1.08, 2.11)**	**0.01**
Sn	**1.07 (1.01, 1.13)**	**0.02**	**1.07 (1.01, 1.13)**	**0.03**
Cr	1.05 (1.01, 1.10)	0.02	1.03 (0.98, 1.08)	0.22
As	1.07 (0.99, 1.16)	0.10	1.05 (0.96, 1.14)	0.30
Fe	**1.36 (1.09, 1.71)**	**0.01**	1.26 (0.99, 1.62)	0.06
Cu	0.95 (0.78, 1.17)	0.64	0.94 (0.76, 1.17)	0.50
Zn	1.12 (0.89, 1.42)	0.32	1.21 (0.93, 1.57)	0.20
Al	0.99 (0.94, 1.04)	0.62	0.98 (0.93, 1.03)	0.35
Sr	1.00 (0.95, 1.06)	0.87	0.99 (0.94, 1.05)	0.73
Sb	1.09 (0.84, 1.43)	0.51	0.98 (0.73, 1.30)	0.79
Be	1.01 (0.98, 1.05)	0.53	1.01 (0.97, 1.05)	0.63
V	1.03 (0.95, 1.12)	0.46	1.04 (0.95, 1.14)	0.63
Mn	1.07 (0.98, 1.17)	0.14	1.04 (0.94, 1.15)	0.41
Co	0.95 (0.84, 1.08)	0.42	0.95 (0.83, 1.09)	0.44
Ni	1.06 (0.98, 1.16)	0.15	1.07 (0.98, 1.16)	0.29
Y	1.00 (0.84, 1.19)	0.98	0.94 (0.78, 1.12)	0.42
Cd	**1.13 (1.01, 1.27)**	**0.04**	1.08 (0.95, 1.23)	0.27
Ce	1.02 (0.94, 1.10)	0.7	0.98 (0.90, 1.07)	0.47
Tl	0.99 (0.92, 1.06)	0.7	0.98 (0.90, 1.05)	0.58
Pb	1.00 (0.96, 1.05)	0.89	0.99 (0.94, 1.04)	0.52

Bold text indicates that the associations were statistically significant at *P* < 0.05. This table presents the analysis results based on the single pollutant model. That is, the correlation of each heavy metal was obtained by treating it (in the form of per log10 increase) as the main exposure variable and placing it alone in a regression model. Al, aluminum; Sb, antimony; As, arsenic; Fe, iron; Cu, copper; Zn, zinc; Sn, tin; Be, beryllium; V, vanadium; Mn, manganese; Sr, strontium; Co, cobalt; Ni, nickel; Y, yttrium; Cd, cadmium; Ce, cerium; Tl, thallium; Pb, lead; Cr, chromium; Se, selenium. ^a^ Models were adjusted for age, sex, BMI, annual income, education, drinking, smoking, physical activity, and serum creatinine.

Se, Sn and Cr were selected for stratified analysis. The association between Sn, Cr and uric acid were stronger in people with non-smoking, compared with those smoking (0.07 mg/dL vs. −0.04 mg/dL, *P* = 0.01, 0.05 mg/dL vs. −0.04 mg/dL, respectively), others stratified analysis had no significant group differences (Supplementary Tables S3, S4).

### 3.3 Association between metal mixtures and SUA or HUA

**Figure 2:**
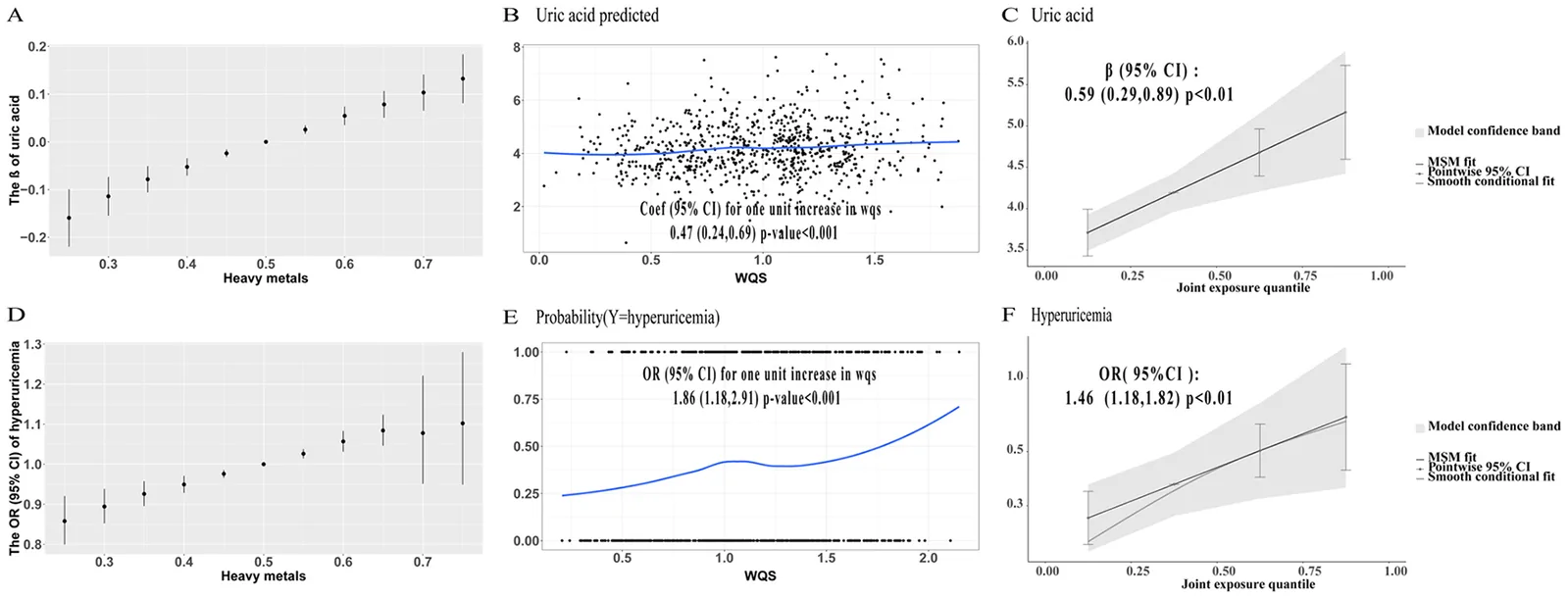
Multi-pollutant modellings of heavy metals on the change of uric acid and the OR of hyperuricemia in all participants (*n* = 1,312). Models were adjusted for age, sex, BMI, serum creatinine, annual income, education, drinking, smoking, and physical activity. **(A–C)** The effect of co-exposure to heavy metals on the change of uric acid in BKMR, positive WQS and qg-comp models, respectively. **(D–F)** The effect of co-exposure to heavy metals on the OR of hyperuricemia in BKMR, WQS and qg-comp models, respectively. BKMR, Bayesian kernel machine regression; WQS, weighted quantile sum; qg-comp, quantile G-computation.

According to BKMR model, we observed positive associations between metal mixtures and SUA as well as HUA odds (Figures 2A, D). The univariate exposure-response plots indicated that positive relationships were observed in Se with SUA and HUA odds, while Sn only had a positive association with SUA (Supplementary Figures S4, S5). PIPs indicated that Se made the most contribution in the joint association (Supplementary Table S5, Figure 1, Supplementary Figure S6). In the WQS regression model assuming a positive correlation between metal mixtures and outcomes, every increase of one quartile in the metal mixture was significantly associated with elevated SUA (β = 0.47; 95% CI: 0.24, 0.69; *P* < 0.001) and higher HUA risk (OR = 1.86; 95% CI: 1.18, 2.91; *P* < 0.001) (Figures 2B, E). Cr made the largest contribution to the positive association with SUA (weight = 0.53), followed by Se (0.13), Sn (0.11), Sr (0,10) and As (0.07). In terms of HUA odds, Cr once again has the greatest influence (0.42), followed by As (0.10), Be (0.09), Sn (0.08), Cd (0.08) and Se (0.06) (Supplementary Table S6, Figure 1, Supplementary Figure S6). However, the metal mixture index was not significantly associated with serum uric acid levels or the risk of hyperuricemia, with Al was the most contributor in both outcome in the WQS regression model assuming a negative correlation between metal mixtures and outcomes (Supplementary Table S7, Supplementary Figure S7). Additionally, qg-comp model found that metal mixtures were positively correlated with SUA levels (β = 0.59; 95% CI: 0.29, 0.89; *P* < 0.01), and with the HUA odds (OR = 1.46; 95% CI: 1.18, 1.82; *P* < 0.01) (Figures 2C, F). Notably, the contribution of Cr and Se on SUA were more significant (weights were 0.37 and 0.11, respectively) (Supplementary Table S8, Figure 1, Supplementary Figure S6). According to the odds of HUA in the qg-comp, Cr was the most contribution (0.32) in positive association, while the weight of Se was only 0.07 (Supplementary Table S8, Figure 1, Supplementary Figure S6). Venn diagram showed that Se was a highly significant metal in all three models (Figure 1).

### 3.4 Associations between metals and TyG

Table 5 shows the correlation between TyG and metals, and a positive correlation was observed in Zn and Se. After adjusting for covariates, each log-unit increase in Zn and Se was associated with 0.14 (95% CI: 0.07, 0.21) and 0.26 (95% CI: 0.18, 0.35) higher TyG index values, respectively.

**Table 5:** Associations of metals with TyG in generalized linear regression models (*n* = 1312).

Heavy metals	Crude model		Adjusted model^a^	
	β **(95% CI)**	***P***	β **(95% CI)**	***P***
Se	**0.33 (0.24, 0.42)**	**< 0.001**	**0.26 (0.18, 0.35)**	**< 0.001**
Sn	0.01 (−0.01, 0.03)	0.37	−0.00 (−0.02, 0.01)	0.66
Cr	−0.01 (−0.03, 0.00)	0.69	−0.02 (−0.03, 0.00)	0.59
As	0.02 (−0.00, 0.05)	0.09	0.02 (0.00, 0.04)	0.06
Fe	**0.07 (0.01, 0.14)**	**0.03**	0.02 (−0.04, 0.09)	0.44
Cu	0.03 (−0.04, 0.09)	0.43	0.04 (−0.02, 0.10)	0.20
Zn	**0.10 (0.03, 0.17)**	**0.01**	**0.14 (0.07, 0.21)**	**< 0.001**
Al	0.00 (−0.02, 0.02)	0.98	−0.00 (−0.02, 0.01)	0.77
[-1pt] Sr	0.01 (−0.00, 0.03)	0.14	0.01 (−0.00, 0.03)	0.07
Sb	0.08 (−0.00, 0.16)	0.06	0.02 (−0.05, 0.10)	0.56
Be	−0.01 (−0.02, 0.00)	0.25	−0.01 (−0.02, 0.00)	0.09
V	0.00 (−0.03, 0.03)	0.94	0.02 (−0.01, 0.04)	0.23
Mn	0.01 (−0.02, 0.03)	0.67	−0.02 (−0.05, 0.01)	0.12
Co	**−0.04 (−0.08, 0.00)**	**0.03**	**−0.04 (−0.08**, **−0.01)**	**0.02**
Ni	−0.01 (−0.03, 0.02)	0.63	−0.01 (−0.03, 0.02)	0.68
Y	0.02 (−0.00, 0.05)	0.15	0.03 (−0.02, 0.08)	0.28
Cd	**0.04 (−0.01, 0.09)**	**< 0.001**	0.02 (−0.01, 0.06)	0.20
Ce	0.05 (0.02, 0.09)	0.65	−0.00 (−0.02, 0.02)	0.91
Tl	0.01 (−0.02, 0.03)	0.08	0.03 (0.00, 0.05)	0.09
Pb	0.02 (−0.00, 0.04)	0.12	−0.02 (−0.04, 0.00)	0.13

TyG, Triglycerides and glucose index. Al, aluminum; Sb, antimony; As, arsenic; Fe, iron; Cu, copper; Zn, zinc; Sn, tin; Be, beryllium; V, vanadium; Mn, manganese; Sr, strontium; Co, cobalt; Ni, nickel; Y, yttrium; Cd, cadmium; Ce, cerium; Tl, thallium; Pb, lead; Cr, chromium; Se, selenium. ^a^Models were adjusted for age, sex, BMI, annual income, education, drinking, smoking, physical activity, and serum creatinine.

### 3.5 Associations of TyG with SUA and the risk of HUA

The study found positive correlations between TyG and SUA levels, as well as the OR of HUA. For each unit increase in TyG, there was a 0.37 mg/dL (95 % CI:0.28, 0.47) change in SUA levels, and an 87 % increase (OR = 1.87, 95% CI:1.53, 2.03) in HUA risk, respectively (Table 6).

**Table 6:** Associations of TyG with uric acid and the risk of hyperuricemia^a^. (n = 1312).

TyG index	Uric acid		Hyperuricemia	
	β (95% CI)	*P* value	OR (95% CI)	*P* value
Crude model	**0.54 (0.45, 0.62)**	**< 0.01**	**2.31 (1.93, 2.80)**	**< 0.01**
Adjusted model^a^	**0.37 (0.28, 0.47)**	**< 0.01**	**1.87 (1.53, 2.03)**	**< 0.01**

TyG, Triglycerides and glucose index. ^a^Models were adjusted for age, sex, BMI, annual income, education, drinking, smoking, physical activity, and serum creatinine based on generalized linear models.

### 3.6 The mediation effects of TyG in the associations of Se with SUA level and HUA odds

**Figure 3:**
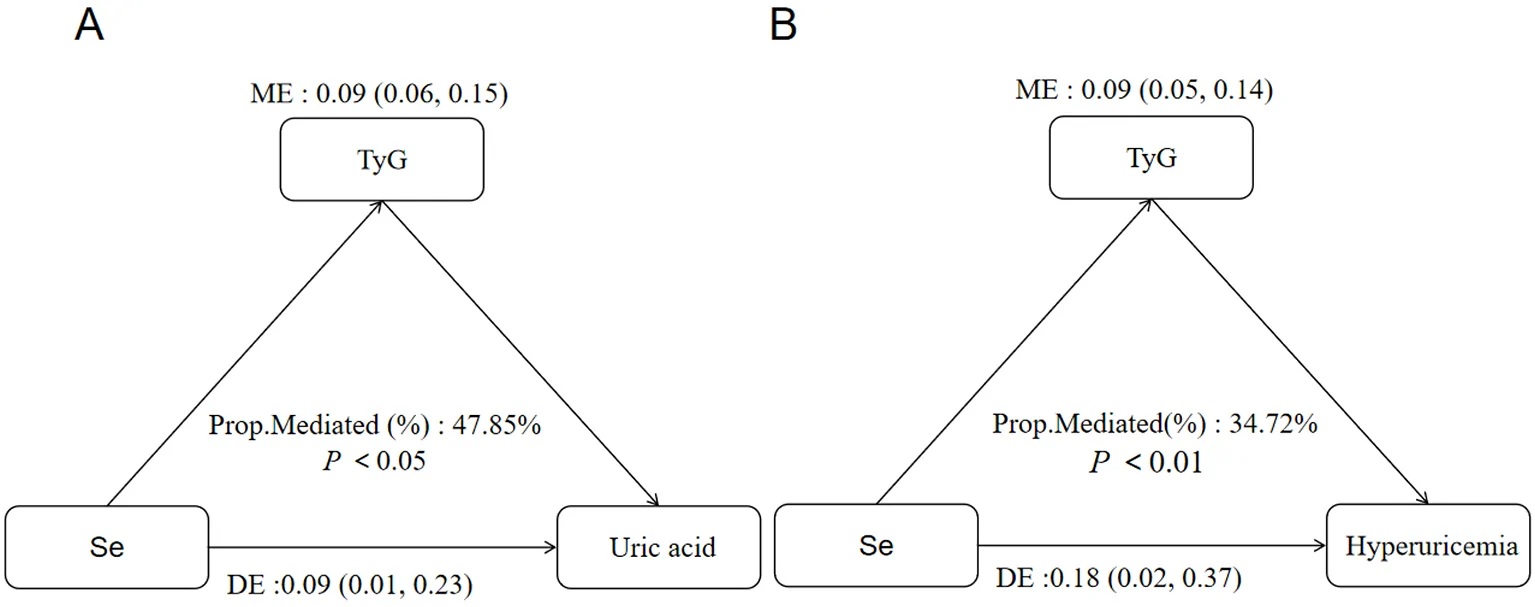
Mediation effects of TyG in the associations of Se with uric acid level and hyperuricemia risk (*n* = 1312). **(A)** Uric acid; **(B)** Hyperuricemia. Models were adjusted for age, sex, BMI, serum creatinine, annual income, education, drinking, smoking, and physical activity. TyG, Triglycerides and glucose index; ME, average causal mediation effects (indirect effect); DE, average direct effects; Se, selenium.

According to the mediator's criterion, only Se met them. Thus, we conducted the mediation analysis of TyG in the associations of Se with SUA and HUA. Furthermore, TyG significantly mediated the associations of plasma Se with SUA level and HUA odds, with the mediated proportions of 47.85% (*P* < 0.05) and 34.72% (*P* < 0.01), respectively (Figure 3, Supplementary Table S9).

### 3.7 Sensitivity analysis

Potential non-linear associations of metals with SUA and HUA odds were shown in Supplementary Tables S10, S11. The results of Se, Sn and Cr were consistent with the main findings. Secondly, the results of the sensitivity analysis using district as a random intercept were consistent with the main findings (Supplementary Tables S12, S13). Third, the results of the model that included hypertension and diabetes as covariates were consistent with those of the main model (Supplementary Tables S14, S15). In addition, the results excluding the participants with CKD were the similar to the main findings (Supplementary Tables S16–S20, Supplementary Figures S8, S9).

### 3.8 Benchmark dose estimation

SUA levels and prevalence of HUA increased with increasing plasma Se concentrations (Supplementary Table S21). Table 7 showed the BMDs and BMDLs of Se EDI for each independent model and model-averaged BMDs at the BMR level of 5 and 10%, respectively. PPP values consistently fell within the 0.05–0.95 acceptability threshold, indicating satisfactory model adequacy across all computational frameworks. At BMR levels of 5% and 10%, the model-averaged BMDLs of Se exposure were 184.62 and 351.71 μg/d for SUA, and 118.70 and 233.12 μg/d for HUA, respectively. The specific parameters of the model are shown in Supplementary Table S22.

**Table 7:** Estimated daily intake of Se BMDs and BMDLs (μg/day) based on BMR of 5% and 10% for SUA and HUA (*n* = 1312).

Model	Weight (%)	PPP	SUA			
			**BMR** = **5%**		**BMR** = **10%**	
			BMD	BMDL	BMD	BMDL
Linear	2.41%	0.46	174.41	130.96	348.81	261.92
Power	40.08%	0.45	377.28	233.63	532.65	397.21
Michaelis Menten	0.01%	0.45	126.68	78.56	274.99	171.99
Hill	11.70%	0.56	387.77	330.31	548.17	477.00
Exponential 2	5.10%	0.45	185.92	144.3	363.19	281.89
Exponential 3	34.73%	0.45	375.83	227.91	534.26	395.39
Exponential 4	0.08%	0.46	166.66	112.99	343.26	235.03
Exponential 5	5.89%	0.44	351.15	210.61	515.16	371.36
Model-average	–	–	369.47	184.62	528.02	351.71
**Model**	**Weight (%)**	**PPP**	**HUA**			
			**BMR**=**5%**		**BMR**=**10%**	
			BMD	BMDL	BMD	BMDL
Logistic	14.22%	0.47	166.41	130.21	319.62	246.79
Probit	15.58%	0.45	156.01	123.12	301.57	235.7
Quantal linear	9.79%	0.50	131.67	85.35	273.33	176.58
Multistage (2nd order)	14.21%	0.50	291.9	176.97	435.74	311.75
Weibull	12.07%	0.48	322.96	163.32	461.95	296.17
LogLogistic	11.52%	0.48	321.39	164.80	450.63	289.6
LogProbit	15.80%	0.45	397.33	299.75	499.97	405.19
Dichotomous Hill	6.82%	0.51	251.46	25.12	392.31	52.91
Model-average	–	–	252.57	118.70	401.44	233.12

SUA, serum uric acid; HUA, hyperuricemia; BMD, benchmark dose; BMDL, benchmark dose lower bound; BMR, benchmark response; PPP, posterior prediction *P*-value.

## 4 Discussion

In this study, we observed positive associations between heavy metal mixture exposure and SUA levels as well as HUA odds, with Se identified as the primary contributor. Notably, insulin resistance was found to partially mediate the associations between plasma selenium concentration and both SUA levels and HUA probability. To our knowledge, this is the first study of insulin resistance's mediating role in the metal-SUA/HUA associations. Furthermore, BMDL of selenium exposure was estimated as 118.70–233.12 μg/d, representing the first application of BMD modeling to establish a POD within a population-based epidemiological investigation in China.

### 4.1 Heavy metal mixture exposure and serum uric acid levels

In the present study, the median plasma concentrations were 49.81 μg/L for Se, 945.23 μg/L for Cu, 882.89 μg/L for Zn, 0.77 μg/L for As, 0.78 μg/L for Pb, 0.56 μg/L for Mn, 0.17 μg/L for Ni, 0.05 μg/L for Tl, and 0.05 μg/L for V. Compared with the Dongfeng–Tongji cohort of middle-to-older-aged Chinese adults (52), which reported median plasma concentrations of 64.22 μg/L for Se, 959.34 μg/L for Cu, 1191.05 μg/L for Zn, 2.03 μg/L for As, 13.10 μg/L for Pb, 4.06 μg/L for Mn, 2.79 μg/L for Ni, 0.14 μg/L for Tl, and 0.69 μg/L for V, our population had a comparable Cu level but lower levels of Se, Zn, As, Pb, Mn, Ni, Tl, and V. In addition, previous studies using plasma or serum reported higher median Se concentrations, including 93.18 μg/L in rural Chinese adults (53) and 126.1 μg/L in NHANES 2011–2016 (54). These differences may be related to geographic region, dietary habits, environmental exposure sources, age and health status, sample matrix, and analytical methods; therefore, cross-study comparisons should be interpreted cautiously.

Accumulating evidence indicates the associations between co-exposure of heavy metals with uric acid homeostasis. However, most of them focused on a limited number of metals (18–20), with only one study examining the association between multiple heavy metals and uric acid levels (10). For instance, a longitudinal study of 3,957 retired employees (averaged age 64 years) from a Chinese automobile manufacturing company demonstrated a positive association between plasma metal mixtures (comprising Sr, V, Ti, Ni, Al, Zn, As, Rb, Mn, Se, Pb, Cu, Ba, Tl, Mo, Co and Sb) and serum uric acid levels (β: 3.17; 95 % CI: 2.42, 3.93) as well as the risk of hyperuricemia incident (OR: 1.47; 95 % CI: 1.23, 1.76) in WQS model, with V and Sr were identified as the primary contributor, respectively (10). Other research had primarily concentrated on the relationships between specific metals and uric acid levels. For example, a community-based cross-sectional study of 3,909 adults revealed a positive relationship between urinary heavy metal mixture (comprising Zn, Fe, Mn, Cu, Pb, Cd, Sb and Ba) and serum uric acid levels in both BKMR and WQS models (WQS index, β: 3.456; 95 % CI: 2.234, 4.678), with Zn identified as the primary contributor (18). Another investigation found positive associations between blood metal mixture (comprising Se, Hg, Mn, Cd, Pb) and uric acid levels (WQS index, β:0.17; 95 % CI: 0.13, 0.20) and the risk of hyperuricemia (WQS index, OR:1.29; 1.47; 95 % CI: 1.16, 1.42) from 2011 to 2018 among 14,871 adults in US, with Pb identified as the primary contributor both in uric acid levels and hyperuricemia (19). These combined effects were consistent with our findings using all three multi-metals models (qg-comp, WQS, and BKMR), but Se was identified as the primary contributor in the present study. The inconsistencies in the findings may be attributable to differences in both the metal species examined and the demographic characteristics of the study populations.

### 4.2 Mechanisms underlying the joint effects of multi-metal exposure on serum uric acid homeostasis

Emerging evidence demonstrates that metal mixture exposure disrupts uric acid homeostasis through synchronized dysregulation of both biosynthetic and excretory pathways. Animal study found that Metal ions such as selenium ions have been shown to enhance xanthine oxidase (XO) activity and expression, thereby upregulating uric acid synthesis in rabbit liver (55). Simultaneously, epidemiological studies have reported a negative association between plasma metal mixtures and kidney function among 1,434 adults in China (56). Similarly, some experimental studies revealed that heavy metals induce renal injury through oxidative stress, inflammatory responses, and disruption of ionic homeostasis (54, 55) (55, 56) (57, 58) (55, 56) (55, 56) (55, 56) (56, 59) (59, 60) (59, 60). This dual-pathway disruption creates a metabolic imbalance wherein increased urate production coincides with diminished renal clearance capacity, promoting pathological accumulation and ultimately leading to hyperuricemia. Further studies are required to elucidate molecular pathways and validate translational implications in human populations exposed to environmental metal mixtures.

### 4.3 Mediation effect of insulin resistance on Se-SUA association

Our findings revealed a novel mechanistic link between selenium (Se) exposure and elevated serum uric acid levels mediated by insulin resistance. Evidence indicates that Se primarily exerts its functions through selenoproteins, some of which, particularly GPx1 overexpression, has been causally associated with impaired insulin signaling in both transgenic murine models and large-scale human studies (*n* = 6,290, age > 20 years in the US) (55, 56, 59). Previous studies have demonstrated that insulin resistance induces increased XO activity (61), the key enzyme in uric acid production (62). Hence, we propose that Se may increase uric acid production through insulin resistance-mediated XO activation. Moreover, another study has reported that insulin resistance disrupts glycolysis, which normally forming pyruvate (63) and then uric acid (64). Elevated Se levels may exacerbate this metabolic shift, further increasing uric acid synthesis. These findings suggest that insulin resistance mediates the Se-uric acid relationship through XO activation and altered glycolytic flux, providing novel insights into the hazardous effects of Se on metabolic health.

### 4.4 Point of departure for Se

Current regulatory frameworks have established selenium exposure limits through various approaches (Supplementary Table S1) (58, 65, 66), yet China lacks population-specific POD for Se based on adverse health outcomes. In previous studies, Selenium and Vitamin E Cancer Prevention Trial (SELECT) identified 330 μg/d as the lowest observed adverse effect level (LOAEL) for Se supplementation (57), which was similar to the no observed adverse effect level (NOAEL) for China populations by European Food Safety Authority (EFSA) (58, 65). Recently, BMD approach is widely adopted in environmental health risk assessments, with its derived BMDL proposed to supersede the NOAEL (67). Our study provides novel evidence that the BMDL for Se-induced hyperuricemia (118.70–233.12 μg/d) is substantially lower than the current Chinese reference value of 255 μg/d (65). The present study carries important regulatory implications, as it: (1) demonstrates the need to reconsider selenium ULs based on metabolic endpoints, (2) establishes the first BMD-derived selenium thresholds for hyperuricemia risk, and (3) provides a methodological framework for future risk assessments of essential trace elements with narrow therapeutic windows.

### 4.5 Strengths and limitations

Our study had several notable advantages. First, we elucidated the novel mediating role of insulin resistance in the metal-uric acid relationship, addressing a critical knowledge gap in understanding the metabolic pathways through which heavy metals influence uric acid homeostasis. Second, our application of benchmark dose modeling provided the first population-based evidence for establishing selenium upper limits (UL) in China. Third, methodologically, we employed a comprehensive approach involving: (1) precise quantification of 20 plasma metals (Se, Al, Sb, As, Fe, Cu, Zn, Sn, Be, V, Mn, Sr, Co, Ni, Y, Cd, Ce, Tl, Pb, and Cr) using ICP-MS, (2) rigorous evaluation through three complementary mixture modeling approaches (BKMR, WQS, and qg-comp), and (3) a community-based sampling design that enhances the generalizability of our findings to the general population. These methodological strengths collectively ensure the robustness and translational value of our conclusions for environmental health policy.

However, our study has some limitations, warranting careful interpretation of the findings. First, as a cross-sectional study, causal relationship cannot be inferred. Second, while we adjusted for multiple confounders, residual confounding may persist from unmeasured variables, such as diet, medications, and genetic factors (68). Third, other environmental pollutants (e.g., pesticides, PFASs) that affect serum uric acid levels were not considered in this study. Fourth, we only measured metal concentrations in plasma, which does not account for cumulative exposure to metals. Future studies should consider incorporating additional biological matrices, such as urine, to provide a more comprehensive understanding of metal exposure and its potential impact on hyperuricemia. Fifth, While TyG is advantageous due to its ease of calculation and predictive capability in certain populations, the absence of the Homeostasis Model Assessment of Insulin Resistance (HOMA-IR)may limit the comparability of our findings with other studies that have utilized this index. Finally, the potential for type I error inflation when interpreting these results. Future studies should incorporate comprehensive exposure assessments using mixture approaches like exposome-wide association studies (ExWAS) to better characterize these complex interactions.

## 5 Conclusion

In summary, this study revealed that exposure to both individual metals and their mixtures were positively associated with uric acid levels and hyperuricemia risk. Insulin resistance might partly mediate the Se-uric acid relationship. The findings give novel evidence to understand the relationship between co-exposure to heavy metals and uric acid levels and its potential mechanisms. Notable a population-based BMDL for Se based on adverse health outcome was established, offering critical data for revising China's upper intake limits. These findings strengthen the rationale for including heavy metals in the growing list of environmental determinants of metabolic disorders. However, further longitudinal and experimental studies are needed to investigate these associations and determine the causality.

## Data Availability

The data analyzed in this study is subject to the following licenses/restrictions: Data will be made available on request. Requests to access these datasets should be directed to Ru-qing Liu, liurq@mail.sysu.edu.cn.
