# Supplementary material for: Selenium predominance in heavy metal mixtures is associated with hyperuricemia in Chinese adults: the role of insulin resistance
Source: Front Public Health. 2026 Aug 27;14:1913054. doi: 10.3389/fpubh.2026.1913054 (PMC13558413; doi:10.3389/fpubh.2026.1913054)
Supplement: Supplementary file 1 [file Supplementary_File_1.docx]

Selenium-Dominated in Heavy Metal Mixtures Promotes Hyperuricemia via Insulin Resistance in Chinese Adults

**Appendix**

**Method S1**

**Method S2**

**Method S3**

**Table S1.** Summary of points of departure and recommended exposure limits of selenium derived from human data.

**Table S2.** Percentile concentrations, limit of detection (LOD), and the detectable ratio of heavy metals quantified among study participants (n = 1312).

**Table S3.** Mean difference (β) and 95% confidence intervals (95% CI) in serum uric acid (mg/dL) per log heavy metals (ng/mL) in generalized linear regression models, by age, sex, BMI according to WHO standard, drinking statue and smoking statue (n = 1312).

**Table S4.** Odd ratio (OR) and 95% confidence intervals (95% CI) for hyperuricemia per log serum heavy metals (ng/mL) in generalized linear regression models, by age, sex, BMI according to WHO standard, drinking statue and smoking statue (n = 1312).

**Table S5.** Posterior inclusion probabilities of each group and individual pollutant into uric acid and hyperuricemia using Bayesian kernel machine regression models (n = 1312).

**Table S6.** Mean weight of individual pollutant into uric acid and hyperuricemia using positive Weighted Quantile Sum (WQS) regression models (n = 1312).

**Table S7.** Mean weight of individual pollutant into uric acid and hyperuricemia using negative Weighted Quantile Sum (WQS) regression models (n = 1312).

**Table S8.** Mean weight of individual pollutant into uric acid and hyperuricemia using quantile-g- computation approach (qg-comp) models (n = 1312).

**Table S9.** Mediation effects of Triglycerides and glucose index (TyG) in the associations of Se with uric acid level and hyperuricemia risk. (n = 1312).

**Table S10.** Adjusted difference (β) and 95% confidence intervals (95% CI) for association between uric acid (mg/dL) and quartiles of heavy metals (n = 1312).

**Table S11.** Adjusted odd ratio (OR) and 95% confidence intervals (95% CI) for association between HUA and quartiles of heavy metals (n = 1312) ^a^.

**Table S12.** Mean difference (β) and 95% confidence intervals (95% CI) in serum uric acid (mg/dL) per log serum heavy metals (ng/mL) in linear mixed effects model. (n=1312)

**Table S13.** Odds ratio (OR) and 95% confidence intervals (95% CI) for hyperuricemia per log heavy metals (ng/mL) in linear mixed effects model. (n=1312)

**Table S14.** Mean difference (β) and 95% confidence intervals (95% CI) in serum uric acid (mg/dL) per log serum heavy metals (ng/mL) in generalized linear regression models. (n=1312)

**Table S15.** Odds ratio (OR) and 95% confidence intervals (95% CI) for hyperuricemia per log heavy metals (ng/mL) in generalized linear regression models. (n=1312)

**Table S16.** Mean difference (β) and 95% confidence intervals (95% CI) in serum uric acid (mg/dL) per log heavy metals (ng/mL) in generalized linear regression models, excluding participants with chronic kidney disease (n = 1154) that may impact serum uric acid.

**Table S17.** Odd ratio (OR) and 95% confidence intervals (95% CI) for hyperuricemia per log heavy metals (ng/mL) in generalized linear regression models, excluding participants with chronic kidney disease (CKD) (n = 1154) that may impact serum uric acid.

**Table S18.** Posterior inclusion probabilities of each group and individual pollutant into uric acid using Bayesian kernel machine regression models, excluding participants with CKD (n=1154) that may impact serum uric acid.

**Table S19.** Mean weight of individual pollutant into uric acid using Weighted Quantile Sum (WQS) regression models, excluding participants with CKD (n = 1154) that may impact serum uric acid.

**Table S20.** Mean weight of individual pollutant into uric acid using quantile-g- computation approach (qg-comp) models, excluding participants with CKD (n = 1154) that may impact serum uric acid.

**Table S21.** SUA levels and prevalence of HUA at different EDI of Se (n=1312).

**Table S22.** Parameters of the model type.

**Figure S1.** Sampling strategy for this study.

**Figure S2.** The direct acyclic graph (DAG) for the association between metals exposure and uric acid.

**Figure S3.** Spearman correlation coefficients among heavy metals in total people (n= 1312).

**Figure S4.** Univariate exposure-response functions of each serum heavy metals (95% CI) and uric acid where the other metals fixed at the median value in BKMR model in total population (n=1312).

**Figure S5.** Univariate exposure-response functions of each serum heavy metals (95% CI) and hyperuricemia where the other metals fixed at the median value in BKMR model in total population (n=1312).

**Figure S6.** Multi-pollutant models showed that the effect of heavy metals on the change of hyperuricemia in total population (n=1312).

**Figure S7.** Total effect and weights of each metal in negative WQS regression.

**Figure S8.** Univariate exposure-response functions of each serum heavy metals (95% CI) and uric acid where the other metals fixed at the median value in BKMR model in participants without CKD (n=1154).

**Figure S9.** Multi-pollutant models showed that the effect of heavy metals on the change of uric acid in participants without CKD (n=1154).

**Method S1**

We have described this process in our previous study(Liu et al., 2024). In brief, 200 μL sample serum was mixed with 2 mL 0.1M formic acid after added 0.5 ng internals standards. The mixture should be thoroughly vortexed. Then, 2 mL 0.1M formic acid and 2mL methanol were added to the Oasis-HLB cartridge column (Waters) successively to activate column. Next, the sample mixture was loaded to the column that was activated, and then washed by 3 mL of 0.1 M formic acid, 6 mL of 50% 0.1 M formic acid/50% methanol, and 1 mL of 1% ammonium hydroxide. The cartridge was dried via vacuum. Then, 2 mL of 1% ammonia acetonitrile was added for elution. Finally, the eluate was reconstituted with a mixture of 70 μL methanol and 30 μL 20 mM ammonium formate.

**Method S2**

Heavy metals in plasma were detected using inductively coupled plasma mass spectrometry (ICP-MS). After the instrument status was stable with relative standard deviation (RSD) less than 5%, the column pipes were cleaned using ultrapure water followed by sample run. Every 20 samples were inserted into a blank sample (2% nitric acid) during detection. The range of heavy metal concentrations in samples was estimated by pretreatment, and different volumes of standard stock solutions of metal elements were taken to prepare standard solutions with different concentration gradients. The correlation coefficients of the standard curves were all higher than 99%. The low, medium and high concentration standard solutions were added to the plasma samples and the recovery at each concentration was measured. According to the measurement results, the average spiked recovery was mainly calculated to be 80%–120%. The blood samples were repeatedly measured 6 times under the same experimental conditions, and the relative standard deviation (RSD) of the peak area ratios of each metal element was calculated to examine the precision of the method (Wang et al., 2017) .

**Method S3**

Serum creatinine was used to calculate the estimated glomerular filtration rate (eGFR). For women, if [Scr](https://www.sciencedirect.com/topics/biochemistry-genetics-and-molecular-biology/creatinine-blood-level) ≤ 0.7 mg/dL, eGFR equals to 144 × (Scr/0.7)^−0.329^ × (0.993)^Age^, and if Scr > 0.7 mg/dL, eGFR equals to 144 × (Scr/0.7)^−1.209^ × (0.993)^Age^. For men, if Scr ≤ 0.9 mg/dL, eGFR equals to 141 × (Scr/0.9) ^−0.411^ × (0.993) ^Age^, and if Scr > 0.9 mg/dL, eGFR equals to 141 × (Scr/0.9) ^−1.209^ × (0.993) ^Age^. In the equation, Scr is expressed as mg/dL and eGFR is expressed as mL/min/1.73 m^2^ (Kong et al., 2013; Su et al., 2022) .

**Table S1.** Summary of points of departure and recommended exposure limits of selenium derived from human data.

| **Reference, year** | **Critical study** | **Limit type** | **POD type, value (μg/d)** | **Endpoint, BMR** | **Final suggested exposure limit**  **(μg/d)** |
| --- | --- | --- | --- | --- | --- |
| (Yang et al., 1987) | (Yang et al., 1987) | RfD | DRI, 41 | The saturation of plasma GPX3, NA | NA |
| (Duffield A.J., 1999) | (Duffield A.J., 1999) | RfD | DRI, 39 | The maximize plasma GPX activity, NA | NA |
| US IOM, 2000 | (Yang et al., 1987) | RfD | EAR, 55 | The saturation of plasma GPX3, NA | NA |
| EFSA, 2000 | (Yang et al., 1989) | UL | NOAEL,850 | Nail alteration or alopecia, NA | 300 |
| (Xia et al., 2010) | (Xia et al., 2010) | RfD | DRI,49 | The saturation plateau of plasma SELENOP levels, NA | NA |
| CNS, 2014 | (Yang et al., 1989) | UL | NOAEL,600 | Nail alteration or alopecia, NA | 400 |
| IOM 2017 | (Yang et al., 1994) | UL | NOAEL,650 | Selenosis, NA | 400 |
| (Feng et al., 2025) | SELECT | UL | LOAEL,330 | Hair loss, NA | 255 |

Abbreviations: POD, point of departure; BMR, benchmark response; RfD, reference dose; EAR, estimated average requirement; DRI, dietary reference intakes; EFSA, European Food Safety Authority; NA, not available; CNS, Chinese Nutrition Society; IOM, Institute of Medicine; UL, tolerable upper intake level; NOAEL, no observed adverse effect level; LOAEL, the lowest observed adverse effect level; SELECT, Selenium and Vitamin E Cancer Prevention Trial.

**Table S2.** Percentile concentrations, limit of detection (LOD), and the detectable ratio of heavy metals quantified among study participants (n = 1,312).

| Heavy metals | Full name | LOD (ng/mL) | Detectable ratio | 5th | 10th | 25th | 50th | 75th | 95th |
| --- | --- | --- | --- | --- | --- | --- | --- | --- | --- |
| Al | Aluminum | 0.133 | 86.68% | 0.094 | 0.094 | 8.413 | 26.590 | 49.983 | 259.450 |
| As | Arsenic | 0.031 | 99.23% | 0.031 | 0.100 | 0.380 | 0.770 | 1.690 | 7.329 |
| Fe | Iron | 0.561 | 99.92% | 351.237 | 433.619 | 569.985 | 790.890 | 1039.425 | 1550.439 |
| Cu | Copper | 0.016 | 100% | 284.297 | 321.318 | 728.473 | 950.475 | 1146.288 | 1506.430 |
| Zn | Zinc | 0.105 | 100% | 305.807 | 350.216 | 700.695 | 883.545 | 1050.635 | 1336.323 |
| Se | Selenium | 0.128 | 100% | 23.744 | 29.184 | 40.995 | 50.020 | 60.233 | 82.284 |
| Sr | Strontium | 0.005 | 98.92% | 0.025 | 2.728 | 17.370 | 29.620 | 41.270 | 63.965 |
| Sn | Tin | 0.010 | 99.92% | 0.035 | 0.035 | 0.080 | 0.200 | 0.413 | 11.700 |
| Sb | Antimony | 0.063 | 99.85% | 1.130 | 1.210 | 1.400 | 1.620 | 1.943 | 3.685 |
| Be | Beryllium | 0.036 | 90.16% | 0.025 | 0.040 | 0.055 | 0.055 | 0.280 | 425.731 |
| V | Vanadium | 0.006 | 100% | 0.014 | 0.014 | 0.014 | 0.050 | 0.160 | 0.420 |
| Mn | Manganese | 0.029 | 99.15% | 0.068 | 0.090 | 0.260 | 0.560 | 1.140 | 3.414 |
| Co | Cobalt | 0.009 | 100% | 0.010 | 0.020 | 0.021 | 0.040 | 0.080 | 0.200 |
| Ni | Nickel | 0.074 | 99.69% | 0.175 | 0.175 | 0.175 | 0.175 | 1.313 | 3.127 |
| Y | Yttrium | 0.003 | 100% | 0.007 | 0.007 | 0.007 | 0.010 | 0.020 | 0.040 |
| Cd | Cadmium | 0.006 | 100% | 0.010 | 0.012 | 0.014 | 0.040 | 0.070 | 0.200 |
| Ce | Cerium | 0.007 | 100% | 0.017 | 0.017 | 0.017 | 0.017 | 0.120 | 0.810 |
| Tl | Thallium | 0.001 | 85.75% | 0.001 | 0.001 | 0.030 | 0.050 | 0.060 | 0.090 |
| Pb | Lead | 0.007 | 100% | 0.016 | 0.016 | 0.016 | 0.780 | 2.000 | 7.125 |
| Cr | Chromium | 0.158 | 100% | 0.373 | 0.373 | 0.373 | 0.373 | 18.288 | 184.300 |

**Table S3.** Mean difference (β) and 95% confidence intervals (95% CI) in serum uric acid (mg/dL) per log heavy metals (ng/mL) in generalized linear regression models, by age, sex, BMI according to WHO standard, drinking statue and smoking statue (n = 1312).

|  | Se | Sn | Cr |
| --- | --- | --- | --- |
| Age | | | |
| ≤ 65 years old  (n=929) | 0.09 (-0.07, 0.26) | **0.06 (0.03, 0.09)** | 0.03 (0.00, 0.06) |
| > 65 years old  (n=383) | 0.14 (-0.12, 0.41) | **0.06 (0.01, 0.11)** | 0.05 (0.00, 0.09) |
| *P* for difference | 0.75 | 0.97 | 0.55 |
| Sex | | | |
| Men (n=515) | 0.14 (-0.09, 0.38) | 0.04 (-0.01, 0.09) | 0.02 (-0.02, 0.06) |
| Women (n=797) | 0.12 (-0.05, 0.30) | **0.06 (0.03, 0.09)** | **0.04 (0.02, 0.07)** |
| *P* for difference | 0.90 | 0.44 | 0.25 |
| BMI | | | |
| Normal  (n=861) | 0.15 (-0.01, 0.31) | **0.05 (0.02, 0.08)** | **0.03 (0.01, 0.06)** |
| Overweight/Obesity (n=451) | 0.20 (-0.10, 0.50) | **0.06 (0.01, 0.12)** | 0.03 (-0.01, 0.08) |
| *P* for difference | 0.77 | 0.58 | 0.99 |
| Drinking status | | | |
| Non-drinking  (n=1128) | 0.13 (-0.02, 0.28) | **0.06 (0.03, 0.09)** | **0.04 (0.02, 0.07)** |
| Drinking  (n=184) | **0.50 (0.06, 0.93)** | 0.02 (-0.07, 0.10) | -0.02 (-0.09, 0.04) |
| *P* for difference | 0.12 | 0.3 | 0.06 |
| Smoking status | | | |
| Non-somking  (n=1092) | **0.17 (0.01, 0.32)** | **0.07 (0.04, 0.10)** | **0.05 (0.02, 0.07)** |
| Smoking  (n=220) | 0.24 (-0.16, 0.65) | -0.04 (-0.12, 0.04) | -0.04 (-0.09, 0.02) |
| *P* for difference | 0.72 | **0.01** | **<0.001** |

Detailed information on the pollutants mentioned is provided in Table S2.

Bold text indicates that the associations were statistically significant at *p*< 0.05.

**Table S4.** Odd ratio (OR) and 95% confidence intervals (95% CI) for hyperuricemia per log serum heavy metals (ng/mL) in generalized linear regression models, by age, sex, BMI according to WHO standard, drinking statue and smoking statue (n = 1312).

|  | Se | Sn | Cr |
| --- | --- | --- | --- |
| Age | | | |
| ≤ 65 years old  (n=929) | 1.56 (1.00, 2.48) | **1.10 (1.02, 1.19)** | 1.04 (0.97, 1.11) |
| > 65 years old  (n=383) | 1.32 (0.79, 2.22) | **1.03 (0.93, 1.14)** | 1.03 (0.95, 1.12) |
| *P* for difference | 0.89 | 0.97 | 1.00 |
| Sex | | | |
| Men (n=515) | 1.80 (1.09, 3.05) | 1.02 (0.92, 1.12) | 0.98 (0.91, 1.06) |
| Women (n=797) | 1.22 (0.77, 1.94) | **1.11 (1.02, 1.20)** | **1.08 (1.01, 1.15)** |
| *P* for difference | 0.75 | 0.95 | 0.95 |
| BMI | | | |
| Normal  (n=861) | 1.32 (0.88, 2.03) | 1.07 (0.99, 1.16) | 1.05 (0.99, 1.12) |
| Overweight/Obesity (n=451) | **1.91 (1.10, 3.37)** | 1.04 (0.95, 1.15) | 1.01 (0.93, 1.09) |
| *P* for difference | 0.75 | 0.99 | 0.98 |
| Drinking status | | | |
| Non-drinking  (n=1128) | 1.42 (0.99, 2.06) | **1.07 (1.01, 1.15)** | 1.06 (1.00, 1.12) |
| Drinking  (n=184) | 2.21 (0.93, 5.48) | 1.02 (0.86, 1.21) | 0.91 (0.79, 1.04) |
| *P* for difference | 0.69 | 0.97 | 0.92 |
| Smoking status | | | |
| Non-somking  (n=1092) | 1.40 (0.99, 2.00) | **1.09 (1.03, 1.17)** | 1.06 (1.00, 1.12) |
| Smoking  (n=220) | 2.12 (0.88, 5.42) | 0.95 (0.80, 1.12) | 0.92 (0.81, 1.04) |
| *P* for difference | 0.77 | 0.92 | 0,93 |

Detailed information on the pollutants mentioned is provided in Table S2.

Bold text indicates that the associations were statistically significant at *p*< 0.05.

**Table S5.** Posterior inclusion probabilities of each group and individual pollutant into uric acid and hyperuricemia using Bayesian kernel machine regression models (n = 1312).

| Heavy metals | Group | Uric acid | |  | Hyperuricemia | |
| --- | --- | --- | --- | --- | --- | --- |
|  |  | groupPIP ^a^ | condPIP ^b^ |  | groupPIP ^a^ | condPIP ^b^ |
| As | 1 | 1.00 | 0.00 |  | 1.00 | 0.00 |
| Al | 2 | 1.00 | 0.00 |  | 0.68 | 0.00 |
| Fe | 2 | 1.00 | 0.00 |  | 0.68 | 0.00 |
| Cu | 2 | 1.00 | 0.00 |  | 0.68 | 0.00 |
| Zn | 2 | 1.00 | 0.00 |  | 0.68 | 0.00 |
| Se | 1 | 1.00 | 1.00 |  | 1.00 | 1.00 |
| Sr | 2 | 1.00 | 0.00 |  | 0.68 | 0.00 |
| Sn | 2 | 1.00 | 1.00 |  | 0.68 | 0.00 |
| Sb | 2 | 1.00 | 0.00 |  | 0.68 | 0.00 |
| Be | 2 | 1.00 | 0.00 |  | 0.68 | 0.00 |
| V | 2 | 1.00 | 0.00 |  | 0.68 | 0.00 |
| Mn | 2 | 1.00 | 0.00 |  | 0.68 | 0.00 |
| Co | 2 | 1.00 | 0.00 |  | 0.68 | 0.33 |
| Ni | 2 | 1.00 | 0.00 |  | 0.68 | 0.00 |
| Y | 2 | 1.00 | 0.00 |  | 0.68 | 0.00 |
| Cd | 2 | 1.00 | 0.00 |  | 0.68 | 0.00 |
| Ce | 2 | 1.00 | 0.00 |  | 0.68 | 0.24 |
| Tl | 2 | 1.00 | 0.00 |  | 0.68 | 0.43 |
| Pb | 2 | 1.00 | 0.00 |  | 0.68 | 0.00 |
| Cr | 2 | 1.00 | 0.00 |  | 0.68 | 0.00 |

Detailed information of pollutants mentioned was provided in Table S2.

Models were adjusted for age, sex, BMI, annual income, education, drinking, smoking, activity, and serum creatinine.

^a^ Posterior inclusion probabilities (PIPs) of each group suggests the relative importance of each group to the outcomes of interest.

^b^ Posterior inclusion probabilities (PIPs) of individual pollutant suggest the relative importance of each exposure within the group to the outcomes of interest.

**Table S6.** Mean weight of individual pollutant into uric acid and hyperuricemia using positive Weighted Quantile Sum (WQS) regression models (n = 1312).

| Heavy metals | Uric acid  weight ^a^ | Hyperuricemia  weight ^a^ |
| --- | --- | --- |
| Cr | **0.53** | **0.42** |
| Se | **0.13** | **0.06** |
| Sn | **0.11** | **0.08** |
| Sr | **0.10** | **0.06** |
| As | **0.07** | **0.10** |
| Be | 0.02 | **0.09** |
| Fe | 0.01 | 0.01 |
| Tl | 0.01 | 0.02 |
| Y | 0.01 | < 0.01 |
| Cd | < 0.01 | **0.08** |
| V | < 0.01 | < 0.01 |
| Zn | < 0.01 | 0.02 |
| Ni | < 0.01 | 0.01 |
| Pb | < 0.01 | 0.03 |
| Cu | < 0.01 | < 0.01 |
| Sb | < 0.01 | < 0.01 |
| Co | < 0.01 | < 0.01 |
| Mn | < 0.01 | 0.02 |
| Ce | < 0.01 | < 0.01 |
| Al | < 0.01 | < 0.01 |

Detailed information of pollutants mentioned was provided in Table S2.

Models were adjusted for age, sex, BMI, annual income, education, drinking, smoking, activity, and serum creatinine.

^a^ Weight of individual pollutant suggests the relative importance to the outcomes of interest.

**Table S7.** Mean weight of individual pollutant into uric acid and hyperuricemia using negative Weighted Quantile Sum (WQS) regression models (n = 1312).

| Heavy metals | Uric acid  weight ^a^ | Hyperuricemia  weight ^a^ |
| --- | --- | --- |
| Al | **0.40** | **0.30** |
| V | **0.26** | 0.03 |
| Y | **0.12** | **0.08** |
| Be | **0.06** | **0.25** |
| Ce | 0.04 | 0.03 |
| Cu | 0.03 | **0.21** |
| Tl | 0.02 | < 0.01 |
| Mn | 0.02 | 0.01 |
| Co | 0.02 | 0.01 |
| As | 0.01 | < 0.01 |
| Sb | 0.01 | 0.03 |
| Cr | 0.01 | 0.02 |
| Fe | < 0.01 | < 0.01 |
| Cd | < 0.01 | < 0.01 |
| Zn | < 0.01 | < 0.01 |
| Pb | < 0.01 | 0.01 |
| Ni | < 0.01 | < 0.01 |
| Se | < 0.01 | < 0.01 |
| Sr | < 0.01 | < 0.01 |
| Sn | < 0.01 | < 0.01 |

Detailed information of pollutants mentioned was provided in Table S2.

Models were adjusted for age, sex, BMI, annual income, education, drinking, smoking, activity, and serum creatinine.

^a^ Weight of individual pollutant suggests the relative importance to the outcomes of interest.

**Table S8.** Mean weight of individual pollutant into uric acid and hyperuricemia using quantile g-computation approach (qg-comp) models (n = 1312).

| Heavy metals | Uric acid | | |  | Hyperuricemia | | |  |
| --- | --- | --- | --- | --- | --- | --- | --- | --- |
|  | direction | weight ^a^ | |  | direction | weight ^a^ | |  |
| Cr | positive | | 0.37 | | positive | | 0.32 | |
| Se | positive | | 0.11 | | positive | | 0.07 | |
| Sr | positive | | 0.10 | | positive | | 0.06 | |
| Ni | positive | | 0.10 | | positive | | 0.16 | |
| As | positive | | 0.09 | | positive | | 0.10 | |
| Sn | positive | | 0.08 | | positive | | 0.06 | |
| Pb | positive | | 0.05 | | positive | | 0.04 | |
| Fe | positive | | 0.03 | | positive | | 0.04 | |
| Tl | positive | | 0.03 | | positive | | 0.04 | |
| Cd | positive | | 0.01 | | positive | | 0.03 | |
| Zn | positive | | 0.01 | | positive | | 0.07 | |
| Be | positive | | 0.01 | | positive | | 0.01 | |
| Ce | negative | | 0.44 | | negative | | 0.48 | |
| Al | negative | | 0.16 | | negative | | 0.14 | |
| Co | negative | | 0.09 | | negative | | 0.10 | |
| Cu | negative | | 0.09 | | negative | | 0.11 | |
| Y | negative | | 0.08 | | negative | | 0.06 | |
| Sb | negative | | 0.08 | | negative | | 0.06 | |
| Mn | negative | | 0.05 | | negative | | 0.05 | |
| V | negative | | 0.01 | | positive | | 0.01 | |

Detailed information of pollutants mentioned was provided in Table S2.

Models were adjusted for age, sex, BMI, annual income, education, drinking, smoking, activity, and serum creatinine.

^a^ weight of individual pollutant suggests the relative importance to the outcomes of interest.

**Table S9.** Mediation effects of Triglycerides and glucose index (TyG) in the associations of Se with uric acid level and hyperuricemia risk. (n = 1312).

| Mediating path | Total effect | Mediation effect | Direct effect | Prop.Mediated |
| --- | --- | --- | --- | --- |
| Se-TyG- Uric acid | 0.18 (0.03, 0.33) | 0.09 (0.06, 0.15) | 0.09 (0.01, 0.23) | 47.85% |
| Se- TyG-Hyperuricemia | 0.27 (0.08, 0.47) | 0.09 (0.05, 0.14) | 0.18 (0.02, 0.37) | 34.72% |

Detailed information of pollutants mentioned was provided in Table S2.

Abbreviations: TyG, Triglycerides and glucose index.

Models were adjusted for age, sex, BMI, annual income, education, drinking, smoking, activity, and serum creatinine.

**Table S10.** Adjusted difference (β) and 95% confidence intervals (95% CI) for association between uric acid (mg/dL) and quartiles of heavy metals (n = 1312) ^a^.

| Heavy metals |  |  | β (95% CI) | | | |
| --- | --- | --- | --- | --- | --- | --- |
|  | Q1 | Q2 | | Q3 | Q4 | *P* for trend |
| Al | Ref | -0.031(-0.185,0.123) | | -0.173(-0.326, -0.020) | -0.130(-0.284,0.023) | **0.04** |
| As | Ref | -0.172 ( -0.324, -0.02) | | 0.062 ( -0.089, 0.213) | 0.047 ( -0.104, 0.198) | 0.22 |
| Fe | Ref | -0.058(-0.210,0.093) | | -0.187(-0.339, -0.035) | 0.010(-0.145,0.165) | 0.23 |
| Cu | Ref | -0.086(-0.241,0.069) | | -0.149(-0.304,0.006) | -0.127(-0.283,0.028) | 0.06 |
| Zn | Ref | -0.240(-0.393, -0.087) | | -0.060(-0.216,0.095) | -0.048(-0.203,0.106) | 0.09 |
| Se | Ref | -0.065(-0.216,0.085) | | 0.095(-0.056,0.247) | **0.223(0.068,0.378)** | **0.04** |
| Sr | Ref | 0.017(-0.135,0.169) | | 0.020(-0.136,0.175) | 0.138(-0.016,0.293) | 0.31 |
| Sn | Ref | 0.124(-0.027,0.275) | | 0.143(-0.008,0.295) | **0.343(0.190,0.496)** | **< 0.01** |
| Sb | Ref | -0.050(-0.201,0.101) | | 0.050(-0.102,0.203) | -0.095(-0.250,0.059) | 0.6 |
| Be | Ref | **0.161(0.010,0.313)** | | -0.023(-0.175,0.128) | 0.117(-0.035,0.269) | 0.51 |
| V | Ref | -0.060(-0.251,0.132) | | 0.045(-0.095,0.186) | -0.039(-0.179,0.100) | 0.83 |
| Mn | Ref | 0.102(-0.049,0.253) | | 0.050(-0.105,0.206) | 0.032(-0.122,0.186) | 0.53 |
| Co | Ref | -0.024(-0.176,0.129) | | 0.020(-0.124,0.163) | -0.057(-0.210,0.095) | 0.63 |
| Ni | Ref | 0.066(-0.086,0.219) | | -0.138(-0.290,0.015) | 0.086(-0.068,0.239) | 0.6 |
| Y | Ref | -0.031(-0.162,0.100) | | 0.003(-0.156,0.161) | -0.185(-0.376,0.005) | 0.1 |
| Cd | Ref | 0.039(-0.105,0.183) | | 0.112(-0.047,0.271) | -0.000(-0.155,0.154) | 0.75 |
| Ce | Ref | 0.064(-0.091,0.219) | | -0.084(-0.237,0.070) | -0.021(-0.177,0.135) | 0.28 |
| Tl | Ref | -0.060(-0.197,0.076) | | -0.030(-0.189,0.130) | -0.026(-0.182,0.130) | 0.52 |
| Pb | Ref | -0.070(-0.225,0.085) | | -0.065(-0.210,0.080) | 0.052(-0.095,0.200) | 0.58 |
| Cr | Ref | **0.207(0.054,0.359)** | | **0.219(0.067,0.371)** | **0.328(0.173,0.484)** | **< 0.01** |

Detailed information on the pollution mentioned is provided in Table S2.

Abbreviations: CI, confidence interval; Q1: quantile 1 (≤25th); Q2: quantile 2 (25th~50th); Q3: quantile 3 (50th ~75th); Q4: quantile 4 (≥75th).

Bold text indicates that the associations were statistically significant at *p*< 0.05.

^a^ Models were adjusted for age, sex, BMI, annual income, education, drinking, smoking, physical activity, and serum creatinine.

**Table S11.** Adjusted odd ratio (OR) and 95% confidence intervals (95% CI) for association between HUA and quartiles of heavy metals (n = 1312) ^a^.

| Heavy metals |  |  | OR (95% CI） | | | | |
| --- | --- | --- | --- | --- | --- | --- | --- |
|  | Q1 | | | Q2 | Q3 | Q4 | *P* for trend |
| Al | Ref | | | 0.981(0.700,1.375) | 0.759(0.538,1.067) | 0.803(0.572,1.124) | 0.13 |
| As | Ref | | | 0.777 (0.552, 1.093) | 1.144 (0.822, 1.592) | 1.363 (0.982, 1.894) | 0.14 |
| Fe | Ref | | | 0.899(0.642,1.259) | 0.819(0.582,1.150) | 1.196(0.851,1.684) | 0.81 |
| Cu | Ref | | | 0.921(0.654,1.295) | 0.847(0.601,1.194) | 0.907(0.644,1.278) | 0.42 |
| Zn | Ref | | | **0.612(0.432,0.864)** | 1.028(0.728,1.450) | 1.128(0.803,1.585) | 0.56 |
| Se | Ref | | | **0.677(0.477,0.957)** | 1.094(0.781,1.533) | **1.466(1.044,2.063)** | 0.51 |
| Sr | Ref | | | 0.880(0.626,1.238) | 1.042(0.739,1.470) | 1.301(0.926,1.830) | 0.57 |
| Sn | Ref | | | 1.373(0.978,1.931) | 1.346(0.957,1.897) | **1.598(1.134,2.259)** | **0.01** |
| Sb | Ref | | | 0.928(0.663,1.300) | 1.096(0.784,1.532) | 0.896(0.637,1.261) | 0.82 |
| Be | Ref | | | 1.449(1.037,2.031) | 1.017(0.725,1.427) | 1.163(0.828,1.634) | 0.85 |
| V | Ref | | | 0.915(0.596,1.395) | 1.028(0.751,1.404) | 1.092(0.803,1.484) | 0.51 |
| Mn | Ref | | | 1.323(0.947,1.850) | 1.170(0.828,1.653) | 1.038(0.736,1.465) | 0.56 |
| Co | Ref | | | 1.059(0.758,1.480) | 0.974(0.711,1.335) | 0.844(0.601,1.182) | 0.34 |
| Ni | Ref | | | 1.348(0.961,1.894) | 0.967(0.685,1.366) | **1.441(1.027,2.027)** | 0.25 |
| Y | Ref | | | 0.936(0.700,1.250) | 1.148(0.811,1.623) | 0.762(0.497,1.158) | 0.4 |
| Cd | Ref | | | 1.148(0.833,1.584) | **1.432(1.009,2.032)** | 1.123(0.795,1.586) | 0.3 |
| Ce | Ref | | | 1.245(0.885,1.754) | 0.987(0.701,1.390) | 1.081(0.765,1.528) | 0.59 |
| Tl | Ref | | | 0.941(0.695,1.274) | 1.015(0.713,1.441) | 1.161(0.827,1.629) | 0.76 |
| Pb | Ref | | | 0.889(0.630,1.252) | 0.775(0.560,1.070) | 1.010(0.732,1.393) | 0.68 |
| Cr | Ref | | | 1.280(0.907,1.811) | **1.448(1.031,2.036)** | **1.485(1.049,2.107)** | 0.19 |

Detailed information on the pollution mentioned is provided in Table S2.

Abbreviations: CI, confidence interval; Q1: quantile 1 (≤25th); Q2: quantile 2 (25th~50th); Q3: quantile 3 (50th ~75th); Q4: quantile 4 (≥75th).

Bold text indicates that the associations were statistically significant at *p*< 0.05.

^a^ Models were adjusted for age, sex, BMI, annual income, education, drinking, smoking, physical activity, and serum creatinine.

**Table S12.** Mean difference (β) and 95% confidence intervals (95% CI) in serum uric acid (mg/dL) per log serum heavy metals (ng/mL) in linear mixed effects model. (n=1312)

| Heavy metals | β (95% CI） | | |
| --- | --- | --- | --- |
|  | Adjusted Model^a^ | *P* values |  |
| Al | 0.01 (-0.03, 0.02) | 0.74 |  |
| As | 0.09 (-0.05, 0.13) | 0.09 |  |
| Fe | 0.14 (-0.03, 0.25) | 0.06 |  |
| Cu | -0.01 (-0.11, 0.09) | 0.86 |  |
| Zn | 0.05 (-0.07, 0.17) | 0.41 |  |
| **Se** | **0.17 (0.02, 0.31)** | **0.02** |  |
| Sr | -0.01 (-0.03, 0.02) | 0.58 |  |
| **Sn** | **0.03 (0.00, 0.07)** | **0.04** |  |
| Sb | 0.04 (-0.10, 0.17) | 0.61 |  |
| Be | 0.00 (-0.02, 0.02) | 0.75 |  |
| V | -0.02 (-0.07, 0.02) | 0.31 |  |
| Mn | 0.04 (-0.01, 0.09) | 0.08 |  |
| Co | -0.02 (-0.09, 0.04) | 0.49 |  |
| Ni | 0.02 (-0.02, 0.07) | 0.31 |  |
| Y | -0.00 (-0.09, 0.09) | 0.98 |  |
| Cd | 0.05 (-0.01, 0.11) | 0.09 |  |
| Ce | -0.01 (-0.05, 0.03) | 0.76 |  |
| Tl | 0.02 (-0.02, 0.06) | 0.27 |  |
| Pb | 0.01 (-0.02, 0.05) | 0.45 |  |
| Cr | 0.02 (-0.01, 0.04) | 0.19 |  |

Detailed information of pollutants mentioned was provided in Table S2.

^a^ Models were adjusted for age, sex, BMI, annual income, education, drinking, smoking, physical activity, and serum creatinine.

**Table S13.** Odds ratio (OR) and 95% confidence intervals (95% CI) for hyperuricemia per log heavy metals (ng/mL) in linear mixed effects model. (n=1312)

| Heavy metals | OR (95% CI） | |
| --- | --- | --- |
|  | Adjusted model^a^ | *P* values |
| Al | 1.00 (0.95, 1.05) | 0.99 |
| As | 1.22 (1.00, 1.34) | 0.05 |
| Fe | 1.41 (1.00, 1.82) | 0.06 |
| Cu | 1.08 (0.87, 1.35) | 0.49 |
| **Zn** | **1.33 (1.02, 1.72)** | **0.03** |
| **Se** | **1.50 (1.09, 2.07)** | **0.01** |
| Sr | 0.97 (0.91, 1.03) | 0.30 |
| Sn | 1.02 (0.95, 1.09) | 0.65 |
| Sb | 1.11 (0.83, 1.50) | 0.49 |
| Be | 0.99 (0.95, 1.03) | 0.49 |
| V | 1.02 (0.93, 1.12) | 0.66 |
| Mn | 1.06 (0.96, 1.17) | 0.25 |
| Co | 0.95 (0.82, 1.09) | 0.44 |
| Ni | 1.11 (1.01, 1.21) | 0.03 |
| Y | 1.03 (0.86, 1.24) | 0.72 |
| Cd | 1.18 (1.03, 1.34) | 0.01 |
| Ce | 1.01 (0.93, 1.11) | 0.74 |
| Tl | 1.07 (0.98, 1.17) | 0.11 |
| Pb | 0.99 (0.92, 1.06) | 0.70 |
| Cr | 1.00 (0.94, 1.06) | 0.95 |

Detailed information of pollutants mentioned was provided in Table S2.

^a^ Models were adjusted for age, sex, BMI, annual income, education, drinking, smoking, physical activity, and serum creatinine.

**Table S14.** Mean difference (β) and 95% confidence intervals (95% CI) in serum uric acid (mg/dL) per log serum heavy metals (ng/mL) in generalized linear regression models. (n=1312)

| Heavy metals | β (95% CI） | | |
| --- | --- | --- | --- |
|  | Adjusted Model^a^ | *P* values |  |
| Al | -0.02 (-0.04, 0.01) | 0.13 |  |
| As | 0.02 (-0.02, 0.06) | 0.24 |  |
| Fe | 0.08 (-0.02, 0.19) | 0.12 |  |
| Cu | -0.07 (-0.17, 0.03) | 0.18 |  |
| Zn | -0.00 (-0.12, 0.11) | 0.94 |  |
| **Se** | **0.18 (0.04, 0.33)** | **0.01** |  |
| Sr | 0.01 (-0.02, 0.04) | 0.43 |  |
| **Sn** | **0.05 (0.03, 0.08)** | **0.01** |  |
| Sb | -0.04 (-0.17, 0.09) | 0.56 |  |
| Be | 0.01 (-0.01, 0.03) | 0.17 |  |
| V | -0.00 (-0.05, 0.04) | 0.89 |  |
| Mn | 0.02 (-0.03, 0.06) | 0.50 |  |
| Co | -0.03 (-0.09, 0.04) | 0.41 |  |
| Ni | 0.00 (-0.04, 0.04) | 0.89 |  |
| Y | -0.05 (-0.14, 0.04) | 0.25 |  |
| Cd | 0.01 (-0.05, 0.07) | 0.74 |  |
| Ce | -0.02 (-0.06, 0.02) | 0.35 |  |
| Tl | -0.02 (-0.05, 0.02) | 0.38 |  |
| Pb | 0.00 (-0.02, 0.03) | 0.92 |  |
| **Cr** | **0.03 (0.01, 0.05)** | **0.01** |  |

Detailed information of pollutants mentioned was provided in Table S2.

^a^ Models were adjusted for hypertension, dyslipidemia, diabetes, age, sex, BMI, annual income, education, drinking, smoking, physical activity, and serum creatinine.

**Table S15.** Odds ratio (OR) and 95% confidence intervals (95% CI) for hyperuricemia per log heavy metals (ng/mL) in generalized linear regression models. (n=1312)

| Heavy metals | OR (95% CI） | |
| --- | --- | --- |
|  | Adjusted model^a^ | *P* values |
| Al | 0.98 (0.93, 1.03) | 0.35 |
| As | 1.08 (1.00, 1.17) | 0.07 |
| Fe | 1.29 (1.02, 1.66) | 0.04 |
| Cu | 1.00 (0.81, 1.25) | 0.97 |
| Zn | 1.22 (0.95, 1.58) | 0.13 |
| **Se** | **1.55 (1.13, 2.15)** | **0.01** |
| Sr | 1.00 (0.95, 1.06) | 0.99 |
| Sn | 1.06 (1.00, 1.12) | 0.06 |
| Sb | 0.99 (0.74, 1.31) | 0.96 |
| Be | 1.00 (0.96, 1.04) | 0.95 |
| V | 1.05 (0.96, 1.15) | 0.29 |
| Mn | 1.02 (0.93, 1.13) | 0.64 |
| Co | 0.94 (0.83, 1.08) | 0.40 |
| Ni | 1.07 (0.98, 1.17) | 0.13 |
| Y | 0.95 (0.79, 1.14) | 0.59 |
| Cd | 1.10 (0.97, 1.25) | 0.15 |
| Ce | 0.99 (0.92, 1.08) | 0.90 |
| Tl | 1.01 (0.93, 1.09) | 0.89 |
| Pb | 0.99 (0.94, 1.04) | 0.58 |
| Cr | 1.03 (0.98, 1.08) | 0.30 |

Detailed information of pollutants mentioned was provided in Table S2.

^a^ Models were adjusted for hypertension, dyslipidemia, diabetes, age, sex, BMI, annual income, education, drinking, smoking, physical activity, and serum creatinine.

**Table S16.** Mean difference (β) and 95% confidence intervals (95% CI) in serum uric acid (mg/dL) per log serum heavy metals (ng/mL) in generalized linear regression models, excluding participants with chronic kidney disease (n = 1154) that may impact serum uric acid.

| Heavy metals | β (95% CI） | | |
| --- | --- | --- | --- |
|  | Adjusted Model^a^ | *P* values |  |
| Al | -0.02 (-0.04, 0.01) | 0.16 |  |
| As | 0.03 (-0.01, 0.07) | 0.20 |  |
| Fe | 0.09 (-0.02, 0.19) | 0.11 |  |
| Cu | -0.08 (-0.18, 0.03) | 0.15 |  |
| Zn | -0.02 (-0.14, 0.10) | 0.77 |  |
| Se | **0.11 (0.04, 0.26)** | **0.03** |  |
| Sr | 0.00 (-0.03, 0.03) | 0.92 |  |
| Sn | **0.05 (0.03, 0.08)** | **<0.01** |  |
| Sb | 0.00 (-0.13, 0.14) | 0.97 |  |
| Be | 0.02 (-0.00, 0.04) | 0.06 |  |
| V | -0.01 (-0.06, 0.03) | 0.57 |  |
| Mn | 0.05 (0.00, 0.09) | 0.05 |  |
| Co | -0.01 (-0.07, 0.06) | 0.81 |  |
| Ni | 0.02 (-0.02, 0.06) | 0.42 |  |
| Y | -0.02 (-0.11, 0.07) | 0.63 |  |
| Cd | 0.02 (-0.04, 0.09) | 0.44 |  |
| Ce | -0.01 (-0.05, 0.03) | 0.57 |  |
| Tl | -0.03 (-0.07, 0.01) | 0.10 |  |
| Pb | 0.01 (-0.02, 0.04) | 0.45 |  |
| Cr | **0.03 (0.01, 0.06)** | **<0.01** |  |

Detailed information of pollutants mentioned was provided in Table S2.

^a^ Models were adjusted for age, sex, BMI, annual income, education, drinking, smoking, physical activity, and serum creatinine.

**Table S17.** Odd ratio (OR) and 95% confidence intervals (95% CI) for hyperuricemia per log serum heavy metals (ng/mL) in generalized linear regression models, excluding participants with chronic kidney disease (n = 1154) that may impact serum uric acid.

| Heavy metals | OR (95% CI） | |
| --- | --- | --- |
|  | Adjusted model^a^ | *P* values |
| Al | 0.98 (0.92, 1.04) | 0.47 |
| As | 1.09 (1.00, 1.19) | 0.06 |
| Fe | **1.39 (1.06, 1.85)** | **0.02** |
| Cu | 0.98 (0.77, 1.26) | 0.90 |
| Zn | 1.22 (0.91, 1.64) | 0.19 |
| Se | **1.50 (1.04, 2.19)** | **0.03** |
| Sr | 0.98 (0.92, 1.05) | 0.55 |
| Sn | 1.07 (1.00, 1.15) | 0.05 |
| Sb | 1.15 (0.83, 1.59) | 0.40 |
| Be | 1.01 (0.97, 1.05) | 0.68 |
| V | 1.05 (0.95, 1.16) | 0.36 |
| Mn | 1.09 (0.98, 1.21) | 0.11 |
| Co | 1.01 (0.87, 1.17) | 0.89 |
| Ni | 1.10 (1.00, 1.21) | 0.06 |
| Y | 1.05 (0.86, 1.28) | 0.61 |
| Cd | **1.17 (1.01, 1.35)** | **0.03** |
| Ce | 1.02 (0.93, 1.12) | 0.65 |
| Tl | 0.97 (0.89, 1.06) | 0.56 |
| Pb | 1.02 (0.96, 1.09) | 0.45 |
| Cr | 1.04 (0.98, 1.10) | 0.17 |

Detailed information of pollutants mentioned was provided in Table S2.

^a^ Models were adjusted for age, sex, BMI, annual income, education, drinking, smoking, physical activity, and serum creatinine.

**Table S18.** Posterior inclusion probabilities of each group and individual pollutant into uric acid using Bayesian kernel machine regression models, excluding participants with CKD (n=1154) that may impact serum uric acid.

| Heavy metals | Group | groupPIP ^a^ | condPIP ^b^ |
| --- | --- | --- | --- |
| As | 1 | 1.00 | 0.03 |
| Al | 2 | 1.00 | 0.00 |
| Fe | 2 | 1.00 | 0.00 |
| Cu | 2 | 1.00 | 0.00 |
| Zn | 2 | 1.00 | 0.00 |
| Se | 1 | 1.00 | 0.97 |
| Sr | 2 | 1.00 | 0.00 |
| Sn | 2 | 1.00 | 0.84 |
| Sb | 2 | 1.00 | 0.00 |
| Be | 2 | 1.00 | 0.00 |
| V | 2 | 1.00 | 0.00 |
| Mn | 2 | 1.00 | 0.00 |
| Co | 2 | 1.00 | 0.00 |
| Ni | 2 | 1.00 | 0.00 |
| Y | 2 | 1.00 | 0.00 |
| Cd | 2 | 1.00 | 0.00 |
| Ce | 2 | 1.00 | 0.00 |
| Tl | 2 | 1.00 | 0.00 |
| Pb | 2 | 1.00 | 0.00 |
| Cr | 2 | 1.00 | 0.16 |

Detailed information of pollutants mentioned was provided in Table S2.

Models were adjusted for age, sex, BMI, annual income, education, drinking, smoking, activity, and serum creatinine.

^a^ Posterior inclusion probabilities (PIPs) of each group suggests the relative importance of each group to the outcomes of interest.

^b^ Posterior inclusion probabilities (PIPs) of individual pollutant suggest the relative importance of each exposure within the group to the outcomes of interest.

**Table S19.** Mean weight of individual pollutant into uric acid using Weighted Quantile Sum (WQS) regression models, excluding participants with CKD (n = 1154) that may impact serum uric acid.

| Heavy metals | Without CKD  weight ^a^ |
| --- | --- |
| Cr | **0.63** |
| As | **0.15** |
| Se | **0.07** |
| Ni | 0.04 |
| Sr | 0.04 |
| Be | 0.03 |
| Fe | 0.01 |
| V | 0.01 |
| Tl | 0.01 |
| Cd | 0.01 |
| Sn | < 0.01 |
| Pb | < 0.01 |
| Cu | < 0.01 |
| Al | < 0.01 |
| Y | < 0.01 |
| Zn | < 0.01 |
| Sb | < 0.01 |
| Ce | < 0.01 |
| Mn | < 0.01 |
| Co | < 0.01 |

Detailed information of pollutants mentioned was provided in Table S2.

Models were adjusted for age, sex, BMI, annual income, education, drinking, smoking, activity, and serum creatinine.

^a^ Weight of individual pollutant suggests the relative importance to the outcomes of interest.

**Table S20.** Mean weight of individual pollutant into uric acid using quantile-g- computation approach (qg-comp) models, excluding participants with CKD (n = 1154) that may impact serum uric acid.

| Heavy metals | Without CKD  direction weight ^a^ | |  |
| --- | --- | --- | --- |
| Cr | positive | 0.40 | |
| Ni | positive | 0.15 | |
| Se | positive | 0.10 | |
| Sr | positive | 0.08 | |
| As | positive | 0.06 | |
| Sn | positive | 0.06 | |
| Tl | positive | 0.03 | |
| Mn | positive | 0.03 | |
| Fe | positive | 0.02 | |
| Zn | positive | 0.02 | |
| Cd | positive | 0.01 | |
| Y | positive | 0.01 | |
| Pb | positive | 0.01 | |
| Ce | negative | 0.51 | |
| Co | negative | 0.17 | |
| Al | negative | 0.17 | |
| Sb | negative | 0.06 | |
| Cu | negative | 0.06 | |
| V | negative | 0.02 | |
| Be | negative | < 0.01 | |

Detailed information of pollutants mentioned was provided in Table S2.

Models were adjusted for age, sex, BMI, annual income, education, drinking, smoking, activity, and serum creatinine.

^a^ weight of individual pollutant suggests the relative importance to the outcomes of interest.

**Table S21.** SUA levels and prevalence of HUA at different EDI of Se. (n=1312)

| EDI, μg/d | All subjects | SUA | | HUA | |
| --- | --- | --- | --- | --- | --- |
|  |  | mean ± SD | *P*-value ^a^ | Cases (%) | *P*-value ^a^ |
| 0-177.99 | 132 | 4.14 ±1.12 | <0.001 | 56 (42.4%) | <0.001 |
| >177.99-257.91 | 132 | 4.21 ±1.15 |  | 46 (34.8%) |  |
| >257.91-298.21 | 130 | 3.88 ±1.00 |  | 39 (30.0%) |  |
| >298.21-332.08 | 132 | 3.84 ±1.08 |  | 42 (31.8%) |  |
| >332.08-365.43 | 130 | 3.93 ±1.07 |  | 35 (26.9%) |  |
| >365.43-401.62 | 131 | 3.97 ±1.02 |  | 47 (35.9%) |  |
| >401.62-443.43 | 131 | 4.06 ±1.10 |  | 66 (50.4%) |  |
| >443.43-501.01 | 131 | 4.35 ±1.11 |  | 53 (40.5%) |  |
| >501.01-626.78 | 131 | 4.15 ±1.06 |  | 63 (48.1%) |  |
| >626.78 | 132 | 4.42 ±1.10 |  | 72 (54.5%) |  |

^a^ *P*-value was calculated by Chi-square linear trend test.

Abbreviation: SUA, serum uric acid; HUA, hyperuricemia; EDI, estimated daily intak

**Table S22.** Parameters of the model type.

| SUA | | HUA | |
| --- | --- | --- | --- |
| Model type | Formula | Model type | Formula |
| Linear | $f(dose)=a+b\times dose$ | Logistic | $f\left( \text{dose} \right)=\frac{1}{1+e^{-a-b\times\text{dose}}}$ |
| Power | $f\left( \text{dose} \right)=a+b\times\text{dose}^{g}$ | Probit | $f\left( \text{dose} \right)=\Phi\left( a+b\times\text{dose} \right)$ |
| Michaelis Menten | $f\left( \text{dose} \right)=a+\frac{b\times\text{dose}}{c+\text{dose}}$ | Quantal linear | $f\left( \text{dose} \right)=a+\left( 1-a \right)\times\left( 1-e^{-b\times\text{dose}} \right)$ |
| Hill | $f\left( \text{dose} \right)=a+\frac{b\times\text{dose}^{g}}{c^{g}+\text{dose}^{g}}$ | Multistage (2nd order) | $f\left( \text{dose} \right)=a+\left( 1-a \right)\times\left( 1-e^{-b\times\frac{\text{dose}}{1-c\times\text{dose}^{2}}} \right)$ |
| Exponential 2 | $f\left( \text{dose} \right)=a\times e^{b\times\text{dose}}$ | Weibull | $f\left( \text{dose} \right)=a+\left( 1-a \right)\times\left( 1-e^{-c\times\text{dose}^{b}} \right)$ |
| Exponential 3 | $f\left( \text{dose} \right)=a\times e^{b\times\text{dose}^{g}}$ | LogLogistic | $f\left( \text{dose} \right)=a+\frac{\left( 1-a \right)}{1+e^{-c-b\times\log\left( \text{dose} \right)}}$ |
| Exponential 4 | $f\left( \text{dose} \right)=a\times\left[ c-\left( c-1 \right)\times e^{-b\times\text{dose}} \right]$ | LogProbit | $f\left( \text{dose} \right)=a+\left( 1-a \right)\times\Phi\left( c+b\times\log\left( \text{dose} \right) \right)$ |
| Exponential 5 | $f\left( \text{dose} \right)=a\times\left[ c-\left( c-1 \right)\times e^{-\left( b\times\text{dose} \right)^{g}} \right]$ | Dichotomous Hill | $f\left( \text{dose} \right)=a\times g+\frac{a-a\times g}{1+e^{-c-b\times\log\left( \text{dose} \right)}}$ |

Abbreviations: SUA, serum uric acid; HUA, hyperuricemia

We randomly selected three districts (Conghua, Panyu, and Yuexiu) from the 11 administrative districts of Guangzhou.

Inclusion criteria: a) were willing to participate in the survey; b) hadn’t severe disease, such as liver or kidney failure, malignancy, and uremia

1,524 residents randomly selected from those who met the inclusion requirements

Yes

No

Excluded, n=78

Age≥18 years old

Yes

No

Excluded, n=12

Complete basic information

Yes

No

Excluded, n=122

Complete blood samples measurement

Participants for final analysis

N=1,312

**Figure S1.** Sampling strategy for this study.

**Figure S2.** The direct acyclic graph (DAG) for the association between metals exposure and uric acid. Color legend: Green: exposure; Blue: outcome; Red: adjusted variable.

**Figure S3.** Spearman correlation coefficients among heavy metals in total people (n= 1312).

**Figure S4.** Univariate exposure-response functions of each serum heavy metals (95% CI) and uric acid where the other metals fixed at the median value in BKMR model in total population (n=1312). The model was adjusted for age, sex, BMI, serum creatinine, annual income, education, drinking, smoking, and physical activity.

**Figure S5.** Univariate exposure-response functions of each serum heavy metals (95% CI) and hyperuricemia where the other metals fixed at the median value in BKMR model in total population (n=1312). The model was adjusted for age, sex, BMI, serum creatinine, annual income, education, drinking, smoking, and physical activity.

**Figure S6.** Multi-pollutant models showed that the effect of heavy metals on the change of hyperuricemia in total population (n=1312). A. Single metal-exposure effect (95% CI) on hyperuricemia when other metals were fixed at 25th, 50th, and 75th percentile in BKMR models. B. The individual contributor of metals mixture to the change of hyperuricemia in positive WQS regression. C. Estimated weight for the association between exposure to metals mixtures and hyperuricemia. D. Venn diagram for two muti-pollutant models. All models were adjusted for age, sex, BMI, serum creatinine, annual income, education, drinking, smoking, and physical activity.

**Figure S7.** Total effect and weights of each metal in negative WQS regression. A-B, uric acid; C-D, hyperuricemia. Models were adjusted for age, sex, BMI, serum creatinine, annual income, education, drinking, smoking, and physical activity.

**Figure S8.** Univariate exposure-response functions of each serum heavy metals (95% CI) and uric acid where the other metals fixed at the median value in BKMR model in participants without CKD (n=1154). The model was adjusted for age, sex, BMI, serum creatinine, annual income, education, drinking, smoking, and physical activity.

**Figure S9.** Multi-pollutant models showed that the effect of heavy metals on the change of uric acid in participants without CKD (n=1154). Models were adjusted for age, sex, BMI, serum creatinine, annual income, education, drinking, smoking, and physical activity. A-C, The effect of co-exposure to heavy metals on the change of uric acid in BKMR, WQS, and qg-comp models, respectively. D. Single metal-exposure effect (95% CI) on the change of uric acid when other metals were fixed at 25th, 50th, and 75th percentile in BKMR models. E. The individual contributor of metals mixture to the uric acid. F. Estimated weight for the association between exposure to metals mixtures and the uric acid. G. Venn diagram for three muti-pollutant models.

**References:**

Kong, X., Ma, Y., Chen, J., Luo, Q., Yu, X., Li, Y., Xu, J., Huang, S., Wang, L., Huang, W., Wang, M., Xu, G., Zhang, L., Zuo, L., Wang, H., 2013. Evaluation of the Chronic Kidney Disease Epidemiology Collaboration equation for estimating glomerular filtration rate in the Chinese population. Nephrol Dial Transplant. 28**,** 641-51. https://doi.org/10.1093/ndt/gfs491.

Liu, Y., Liang, R., Liu, W., Yu, L., Huang, X., Cao, X., Wang, B., Ma, J., Zhou, M., Chen, W., 2024. Traffic-related heavy metals exposure with serum uric acid and hyperuricemia in general Chinese urban adults: Roles of systemic inflammation. Journal of Environmental Sciences. https://doi.org/10.1016/j.jes.2024.05.026.

Su, F., Zeeshan, M., Xiong, L., Lv, J., Wu, Y., Tang, X., Zhou, Y., Ou, Y., Huang, W., Feng, W., Zeng, X., Dong, G., 2022. Co-exposure to perfluoroalkyl acids and heavy metals mixtures associated with impaired kidney function in adults: A community-based population study in China. Sci Total Environ. 839**,** 156299. https://doi.org/10.1016/j.scitotenv.2022.156299.

Wang, Q., Li, M., Yuan, Y., Li, Y., Liu, Z., Zhou, D., Zou, X., 2017. [Direct determination for 22 kinds of elements in umbilical cord serum by ICP-MS]. Wei Sheng Yan Jiu. 46**,** 772-776.
